# Applications of synchrotron light in seed research: an array of x-ray and infrared imaging methodologies

**DOI:** 10.3389/fpls.2024.1395952

**Published:** 2025-02-17

**Authors:** Paula Ashe, Kaiyang Tu, Jarvis A. Stobbs, James J. Dynes, Miranda Vu, Hamid Shaterian, Sateesh Kagale, Karen K. Tanino, Janitha P. D. Wanasundara, Sally Vail, Chithra Karunakaran, Teagen D. Quilichini

**Affiliations:** ^1^ Aquatic and Crop Resource Development, National Research Council Canada, Saskatoon, SK, Canada; ^2^ Canadian Light Source Inc., Saskatoon, SK, Canada; ^3^ Department of Plant Sciences, College of Agriculture and Bioresources, University of Saskatchewan, Saskatoon, SK, Canada; ^4^ Agriculture and Agri-Food Canada, Saskatoon Research Centre, Saskatoon, SK, Canada; ^5^ Department of Biology, College of Arts and Science, University of Saskatchewan, Saskatoon, SK, Canada

**Keywords:** synchrotron, micro-computed tomography (µCT), mid-infrared spectroscopy, x-ray absorption spectroscopy (XAS), x-ray fluorescence spectroscopy (XRF), spectromicroscopy (SM), seed, embryo

## Abstract

Synchrotron radiation (SR) provides a wide spectrum of bright light that can be tailored to test myriad research questions. SR provides avenues to illuminate structure and composition across scales, making it ideally suited to the study of plants and seeds. Here, we present an array of methodologies and the data outputs available at a light source facility. Datasets feature seed and grain from a range of crop species including *Citrullus* sp. (watermelon), *Brassica* sp. (canola), *Pisum sativum* (pea), and *Triticum durum* (wheat), to demonstrate the power of SR for advancing plant science. The application of SR micro-computed tomography (SR-µCT) imaging revealed internal seed microstructures and their three-dimensional morphologies in exquisite detail, without the need for destructive sectioning. Spectroscopy in the infrared spectrum probed sample biochemistry, detailing the spatial distribution of seed macronutrients such as lipid, protein and carbohydrate in the embryo, endosperm and seed coat. Methods using synchrotron X-rays, including X-ray absorption spectroscopy (XAS) and X-ray fluorescence (XRF) imaging revealed elemental distributions, to spatially map micronutrients in seed subcompartments and to determine their speciation. Synchrotron spectromicroscopy (SM) allowed chemical composition to be resolved at the nano-scale level. Diverse crop seed datasets showcase the range of structural and chemical insights provided by five beamlines at the Canadian Light Source, and the potential for synchrotron imaging for informing plant and agricultural research.

## 1 Introduction

A synchrotron facility functions in the acceleration then re-direction of electrons along a curved path; a process that emits strong radiation (Balerna and Mobilio, 2014). Synchrotron light or synchrotron radiation (SR) spans the electromagnetic spectrum, from low energy infrared, through soft X-rays to high energy hard X-rays, and is extremely bright, with high flux per unit area (Bharti and Goyal, 2019). The characteristic brightness and energy tunability of SR provides an adaptive tool with broad applications in science and medicine. Although applications of SR in medical research are prevalent, this tool has yet to become commonplace in agricultural research. The lag in synchrotron adoption by the life sciences, and particularly plant science research may result from perceived access restrictions to these facilities, and uncertainty among scientists surrounding the applications available and insights possible.

SR provides a unique tool for obtaining vast structural and chemical insights on the inner workings of organisms. In seed-bearing plants, offspring are efficiently packaged in a protected capsule to support independent survival. Seeds consist of three distinct structures, the embryo, endosperm and seed coat, which are intimately linked by physical proximity but differ genetically and biochemically (Xiong et al., 2021). Within a seed, the progeny progresses from a single-celled zygote to an embryo with shoot and root axes that is poised for germination (Ramalho et al., 2021). The developing embryo is surrounded by multinucleated cells of the triploid endosperm that primarily functions as a nutritive tissue (Sabelli and Larkins, 2009; Doll and Ingram, 2022). The abundance of endosperm varies dramatically among species, persisting as a prominent storage tissue in monocotyledonous (monocot) plants including cereals such as wheat, rice and maize, or absent in mature seeds of many dicotyledonous (dicot) plants, including legumes and brassicas (Sabelli and Larkins, 2009). A seed coat forms the outermost casing of a seed and envelops the endosperm and embryo within a hardened exterior. As a seed develops, the seed coat forms at the interface between offspring and mother plant, and later between offspring and the external environment (Radchuk and Borisjuk, 2014; Huss and Gierlinger, 2021). This crucial locale requires seed coats to be multifunctional, including as a conduit for nutrient transfers between generations during early seed development, then as a fortified, often dead, barrier that protects the filial generation and mediates seed dispersal, dormancy and germination. Beyond the crucial function seeds serve in the reproductive success of many plants, seeds and grains serve as a primary source of calories for human and animal diets, supplying diverse nutrient profiles rich in protein, fiber, fats, vitamins, and minerals (Preedy et al., 2010). Thus, the agricultural sector requires vigorous nutrient-dense seeds that meet consumer demands, maintain viability after sustained storage, and yield crops that are productive and stress resilient. For seed research, synchrotron imaging is particularly informative for resolving structural relationships amongst seed tissues to support quality, health and viability assessments, and for mapping compositional data to each seed subcompartment to assess nutrient landscapes and commercial value.

Adoption of synchrotron-based imaging in plant and agricultural research has grown in recent years, but is generally in its infancy (Vijayan et al., 2015; Indore et al., 2022). Research strategies that uncover the internal structural phenotypes and nutritional content of seeds offer opportunities to identify preferred traits and targets for improvement. Here, we showcase an assortment of seed datasets to demonstrate the potential for synchrotron imaging in plant science, using the Canadian Light Source (CLS) in Saskatoon, Canada. Methods presented cover seed preparation options to analyze mature, dry seeds as well as fresh, developing seeds undergoing embryogenesis at five CLS beamlines. Seeds analyzed come from a variety of valued dicot and monocot crops, including oil-rich canola, protein-rich pea, and carbohydrate-rich wheat. We feature CLS beamlines that deploy hard and soft X-rays, deemed widely relevant to life science research and available at synchrotron facilities around the globe. For each beamline, basic capabilities are detailed and examples are provided to demonstrate data output potential. Briefly, seed three-dimensional (3D) structure is analyzed in high resolution with synchrotron-based micro-computed tomography (SR-µCT) at the biomedical imaging and therapy low-energy beamline (BMIT-BM, 12.6 – 40.0 keV spectral range). Seed protein, carbohydrate and lipid macronutrients are spatially mapped across seeds with mid-infrared spectroscopy (MidIR spectroscopy 560-6000 cm^-1^; with and without synchrotron light). Micronutrients, including calcium (Ca), potassium (K), manganese (Mn), magnesium (Mg), cadmium (Cd), zinc (Zn), iron (Fe), and phosphorus (P) are spatially mapped across seed tissues using a combination of hard X-ray fluorescence (XRF) imaging at the Biological X-ray absorption spectroscopy beamline (BioXAS-Imaging, at 5 – 21 keV), and XRF imaging at the soft X-ray microcharacterization beamline (SXRMB, 1.7 – 10 keV). The chemical speciation of elements is examined using X-ray Absorption Near Edge Structure (XANES) spectroscopy, to distinguish distributions of Zn (at BioXAS-Imaging). Fine elemental mapping with soft X-ray spectromicroscopy (SM, 130 – 3000 eV) is used to characterize subcellular distributions of P.

This article uses case studies and select examples to describe methods and to highlight the utility of synchrotron imaging technologies for seed research. While the article presents novel structural and compositional datasets, the article is not intended to describe complete research stories.

Case Study 1: SR-µCT of *Acidovorax citrulli* infected watermelon seeds. Bacterial fruit blotch caused by *Acidovorax citrulli* is a global threat to members of the Cucurbitaceae family such as watermelon and seeds are the primary source of inoculum. There are no known sources of resistance to this pathogen and antibacterial seed treatments have limited efficacy (Burdman and Walcott, 2012; Carvalho et al., 2012). Pathogen localization in the seed is related to the mode of infection and penetration into the embryo occurs with pistil infection (Dutta et al., 2012). SR-µCT provides the unique ability to visualize the extent of pathogen infection and resulting necrotic damage in 3D space. This application of SR-µCT imaging could be useful for testing outcomes of seed disinfestation treatments and for studies on the pathogenesis of plant disease.

Case Study 2: Multi-beamline analysis of the anatomic and compositional changes in pea seed tissues during development. Developing seeds require special consideration for comparison between genotypes and treatments, as developmental staging is critical to make informed conclusions. Destructive seed dissection to visualize the embryo can be used for developmental staging but results in the loss of 3D spatial information. Sectioning for microscopy and the resulting 2D images requires careful orientation of the plane of section to view developing embryos. SR-µCT can provide 3D visualization of the embryo for accurate developmental staging as well as provide cell layer specific information through the seed coat and embryo and volumetric analysis of seed components. This information across multiple pollination days, genotypes and treatments can be used to accurately match samples for subsequent 2D chemometric imaging. This case study provides an example of semi-correlative microscopy of developing seeds across multiple beam lines.

Case Study 3: Phosphorus distribution in high and low phytate canola genotypes. Phytate is the main storage form of P and is a known anti-nutritional factor due to chelation of cationic elements (Thompson, 1990; Madsen and Brinch-Pedersen, 2020). In canola, phytate is stored in globoids inside protein storage vacuoles primarily in the cotyledons. Multiple beamlines were used to interrogate the distribution of protein, lipid and P in canola genotypes differing in their phytate content. This case study is not only an example of cross beam correlative and semi-correlative microscopy, but also serves to demonstrate the use of beamlines with lower energies to better interrogate low atomic weight elements such as P. Limitations and considerations for examining P in biological samples are also discussed.

## 2 Materials and methods

### 2.1 Overview of the Methods

**Figure 1:**
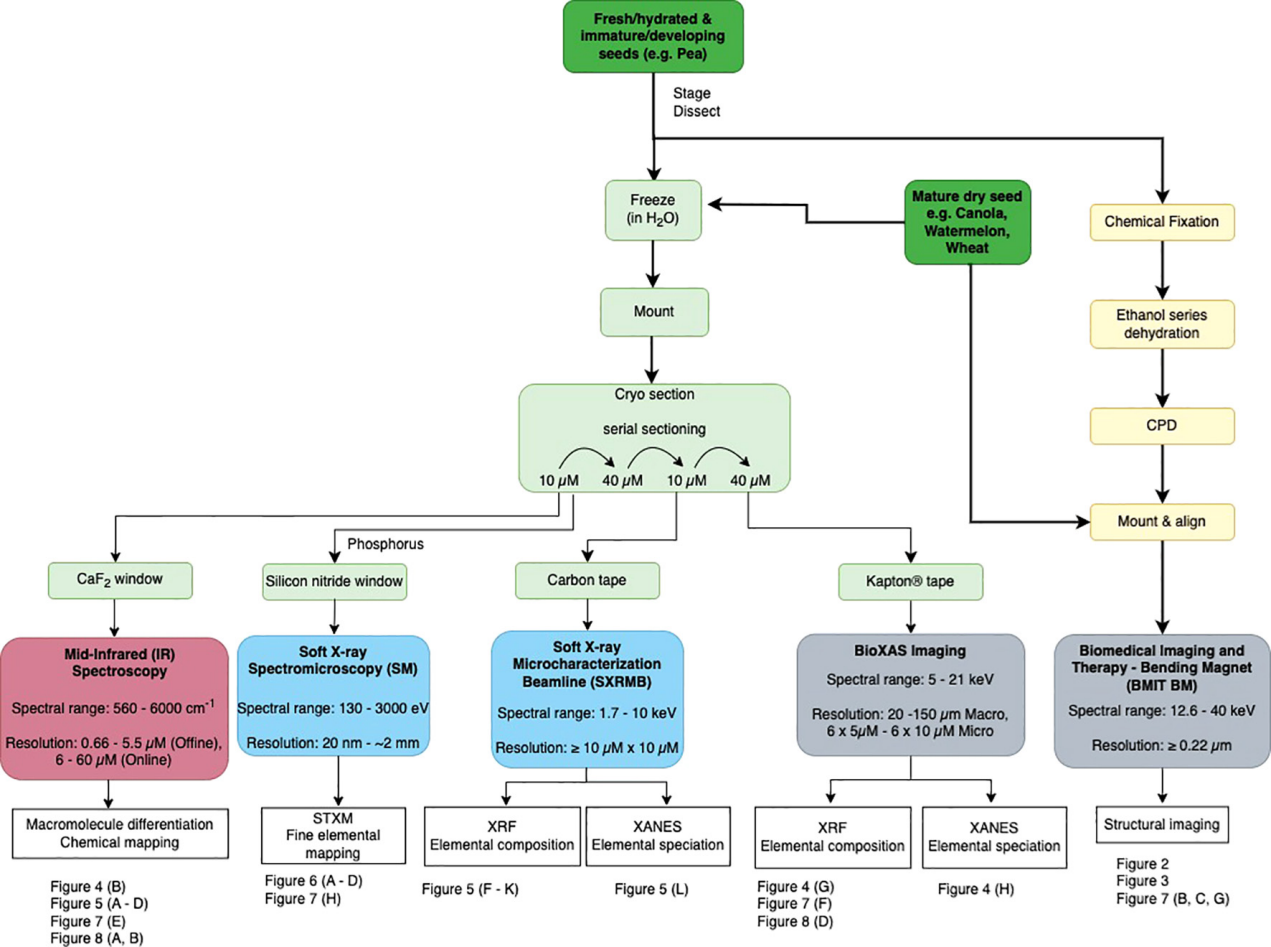
Workflow for imaging seeds with synchrotron light, for five agriculturally relevant beamlines at the Canadian Light Source. Dark green boxes indicate seed sample entry points. Procedural steps (detailed in Methods) to prepare seeds for each beamline are outlined, beginning with specimen input options (dark green), then methodologies to prepare specimens for the BMIT-BM beamline (yellow), and BioXAS-Imaging, SXRMB SM and MidIR spectroscopy beamlines (pale green). Beamlines are colour-coded according to the spectrum of electromagnetic radiation employed; red uses infrared light, blue use soft X-rays, and grey use hard X-rays. Beamline spectral range (in keV) and resolution capabilities are included with beamline titles. Resolutions reflect pixel size options for output data. Data outputs of each beamline, as featured in this study, are summarized in white boxes. Reference examples of the data outputs available are indicated by figure numbers below each beamline.

The methods in our study provide generalized procedures to prepare seeds for synchrotron imaging analysis (**Figure 1**). Given the heterogeneity amongst seeds, and the breadth of research questions that synchrotron light can address, the methodologies describe general workflow recommendations, as detailed protocols that will optimally prepare all specimens for synchrotron-based research are not feasible. The methods are intended to provide a framework for new users to develop, adapt and optimize approaches according to their research needs. We present methods and synchrotron imaging data for diverse crop seeds, known for their unique abundance of oil, proteins or carbohydrates, to demonstrate the potential for implementing these methods in many crop species and experimental pursuits. Methods include recommended approaches for preparing mature seed as well as immature, fresh seed in which embryos are developing. Each beamline used in this study has specific sample preparation requirements. Therefore, we outline key beamline-specific considerations for appropriately preparing materials. Readers are encouraged to consult additional published works employing synchrotron light to study seeds and seed germination, including studies employing XRF imaging (Kim et al., 2006; Iwai et al., 2012; Kopittke et al., 2018; Romeu et al., 2021; van der Ent et al., 2022; Montanha et al., 2022; Ren et al., 2022) and synchrotron X-ray CT approaches (Yamauchi et al., 2012; Rousseau et al., 2015; Le et al., 2019; Legland et al., 2022; Indore et al., 2024). Examples and reviews of seed MidIR spectroscopy can be found in Feng et al., 2020; Hossain et al., 2022; Guendel et al., 2018; Türker-Kaya and Huck, 2017; Yan et al., 2020 and references therein. It is advised that experimental design and methodologies are reviewed in consultation with synchrotron experts prior to initiating new studies, to appropriately tailor protocols according to beamline standards and recommendations.

### 2.2 Seeds

- Pea (*Pisum sativum*) seeds (USDA seed repository Pullman, WA).
- Canola (*Brassica napus*) genotype DH4079, a doubled haploid line obtained from the Swedish spring cultivar, Topas, (Dr. Alison Ferrie, NRC, Saskatoon). Two genotypes were selected from a *Brassica napus* diversity collection based on phytate levels (NAM13; high phytate and NAM45; low phytate) (Ebersbach et al., 2022).
- Wheat (*Triticum durum*) cultivar ‘Pelissier’ seeds (Dr. Raju Soolanayakanahally, Agriculture and Agri-Food Canada, Saskatoon).
- Watermelon (*Citrullus lanatus*) seeds infected with *Acidovorax citrulli*, a seedborne bacterium known to cause bacterial fruit blotch disease (Dr. Greg Welbaum, Virginia Tech) (Eckshtain-Levi et al., 2014).

### 2.3 Preparing seeds for synchrotron-based micro-computed tomography

Plant growth should involve controlled conditions, to remove unintended variations in seed structure and/or composition. For SR-based micro-computed tomography (SR-µCT) imaging (BMIT-BM) mature seeds do not require fixation or drying, and can be directly mounted to the stage and imaged. For SR-µCT imaging of fresh materials including immature seeds spanning embryogenesis, chemical fixation and dehydration are recommended (Section 2.3.1). For chemical imaging (MidIR spectroscopy, BioXAS-Imaging, SXRMB and SM), seeds must be cryo-sectioned and dried on beamline-appropriate substrates (Section 2.4.2).

- For preparing immature seeds, staging flowers as they form can assist in developmental timing decisions.

#### 2.3.1 Chemical fixation and dehydration of immature pea seeds for SR-µCT imaging (BMIT-BM)

- An overview of the chemical fixation protocol recommended for fresh, immature seeds is provided here, with additional protocol details and suggestions provided (See **Supplementary Methods**).
- Use a dissecting stereo microscope and forceps (Dumont #5 forceps, Fine Science Tools; Cedarlane, Burlington, ON) to remove developing seeds from the parent plant without damaging the seed coat. Minimize time from dissection to chemical fixation (for SR-µCT).
- Recommended fixative solution: Glutaraldehyde (25% aqueous glutaraldehyde, Electron Microscopy Sciences; Cedarlane Burlington, ON) diluted to a final concentration of 2% (v/v) in 25 mM PIPES buffer, pH 7.0 (PIPES or 1,4-Piperazinediethanesulfonic acid, MilliporeSigma, Oakville, ON). Prepare fresh fixative solution on the day it will be used.
- To fix, submerge seeds in fixative solution in glass scintillation vials and leave at room temperature for 2 hours or overnight at 4°C. Plant material to fixative ratio should be 1:1000.
- Following fixation, replace with 25 mM PIPES buffer, pH 7.0 without glutaraldehyde, while ensuring samples remain submerged. Repeat 5 times, to ensure fixative has been removed.
- Dehydrate samples in ascending ethanol; 30%, 50%, 70%, 95%, 100% with a minimum 20 minute incubation at room temperature between each exchange. Ethanol (anhydrous absolute ethanol, Greenfield Global, Toronto, ON) solutions can be made with ultrapure water (Synergy UV Water Purification System, MilliporeSigma, Oakville, ON).
- Repeat a minimum of three 100% ethanol exchanges for 20 minutes each. For materials exceeding 1 cm in diameter, it is recommended to lengthen time between exchanges, and to allow seeds to remain in 100% ethanol for a minimum of 48 hours prior to critical point drying.
- Critical point dry the samples using stasis mode (Autosamdri^®^-931 Critical Point Dryer, Tousimis Research Corp, Rockville, MD); 3 cycles with solvent-substituted liquid CO_2_ (as a drying agent). Thoroughly dried samples should have no detectable ethanol odour.
- Store samples in a sealed container away from light, with dust-free desiccant.

#### 2.3.2 Mount seeds for SR-µCT imaging (BMIT-BM)

- Affix seed to a stub (SEM pin stubs, Ted Pella Inc, Redding, CA). Hot glue is well suited to mounting seeds, including critical pointed dried materials. Other mounting substrates such as dental wax or double sided carbon tape are also acceptable. It is critical that the sample is firmly mounted to eliminate motion during scanning.
- Sample can be stored in an SEM stub box (Ted Pella Inc, Redding, CA), or mounted directly on a goniometer head for SR-µCT imaging (see Section 2.5).

### 2.4 Preparation of seed sections for MidIR, BioXAS-imaging, SXRMB and SM

Accessing spatially-resolved internal chemistry typically requires thin cryo-sectioning of biological materials. For all chemical imaging methods described by this study, seed materials could not be chemically fixed or embedded in resin prior to sectioning, as this alters metabolic composition. This is critical for MidIR spectroscopy, however, if only elemental analysis is required (XRF or XANES), sample processing, such as fixation, with careful consideration is possible (for review see Pushie et al., 2022). Ultrapure water is predominantly used as an embedding medium to freeze seeds in preparation for cryosectioning. The cryo-sectioning methods outlined below are for samples embedded in water, where measurements using IR and XRF require clean analysis of the outermost seed layers (e.g. seed coat and aleurone). If analysis is restricted to inner tissues such as the embryo, and/or IR will not be used, direct embedding of seeds into cryo-gel (Pelco cryo-embedding compound; Ted Pella Inc, Redding, CA) or other non-infiltrating media is sufficient.

#### 2.4.1 Cryo-preservation of seeds for cryo-sectioning

- Wear gloves throughout sample preparation. Do not touch samples or substrates as any contamination will impact subsequent analysis.
- Hydrated seed drastically improves the quality of cryo-sections. For mature seed, soaking pretreatment in ultrapure water for ~8 h to overnight is typically sufficient for re-hydration. For fresh seed including immature or green seed, no re-hydration step prior to embedding is required.
- Samples are frozen in water to ensure a hydration sphere around the tissue as this will minimize artifacts in subsequent analysis. Using water as the embedding medium ensures samples can be analyzed by IR. Ultrapure water (Synergy UV Water Purification System, MilliporeSigma, Oakville, Ontario) is recommended.
- Carefully place an isolated seed in a capsule (BEEM^®^ embedding capsules, Electron Microscopy Sciences; Cedarlane Burlington, ON) or 2 mL microfuge tube (Avantor, Mississauga, ON) filled with water. The container should be selected to ensure sufficient space for the seed and at least a thin shell of surrounding water. Minimize time from dissection of seeds to cryo-preservation. Orient the sample to aid subsequent mounting for cryo-sectioning, if possible.
- Flash freeze the sample in liquid nitrogen (in a dewar) and store at -80˚C until cryo-sectioning. To minimize cryodamage to tissues due to the formation of ice crystals during flash freezing, samples may alternatively be frozen at higher temperatures in a slurry of dry ice/isopentane, liquid ethane or propane or under high-pressure. It should be noted that cryoprotectants such as sucrose or glycerol are not suitable as they will interfere with the generated MidIR spectra. Avoid storage exceeding 6 months.

#### 2.4.2 Cryo-sectioning seeds

- Set the cryostat (Leica CM1950 cryostat, Leica Biosystems, Concord, ON) to the desired sectioning temperature and let it equilibrate. Temperature for sectioning will depend on the composition and nature of the sample as well as environmental conditions in the lab (e.g. humidity). A reasonable starting temperature is -20˚C. If the sections stick or roll, decrease the temperature. If the sections crack, increase the temperature. It is recommended to begin with a sacrificial sample to optimize sectioning parameters.
- In the cryostat, use cryo-mounting medium (Pelco cryo-embedding compound, Ted Pella Inc, Redding, CA) to secure the sample to a pre-cooled cryostat chuck.
- Remove the sample from the tube by warming the sides of the tubes until the ice block containing the sample slides out.
- Excess ice may be trimmed away, but a thin layer must be kept to ensure separation between the sample and the mounting medium.
- Equilibrate the mounted sample to the cryostat prior to initiating cryo-sectioning.
- The recommended substrates for collecting cryosections differ according to the intended method of analysis.

Cryosection substrate options include:

      • For MidIR spectroscopy (online and offline), calcium fluoride (CaF_2_) windows (Calcium Fluoride polished windows, 25 mm x 1 mm thick; Crystran, Dorset, UK). Thorough cleaning of windows ensures a clean background for IR measurements, and is crucial anytime a CaF_2_ window is re-used. To clean, rinse with ultrapure water followed sequentially by ethanol, isopropanol, and acetone.

– For BioXAS or SXRMB, Kapton^®^ (Avantor, Mississauga, ON) or carbon tape (Ted Pella Inc, Redding, CA).
– For SM, silicon nitride windows (5 mm frame, 0.5 mm aperture, 200 nm membrane; Ted Pella Inc, Redding, CA).
– Substrates for cryo-sections should be at room temperature to aid section transfers from the cryostat.

- Section into frozen specimen block with 60 – 80 µm steps to approach region of interest, then reduce step size. Inspect sections with a compound microscope (Revolve Echo, San Diego, CA) to ensure region of interest has been reached.

- For MidIR, section at a thickness of 6 to 12 µm and collect sections on a clean CaF_2_ window. Multiple sections can be collected on a single window.

– Section thickness should be decided for the sample and resolution required. 6 µm is ideal, but for some seeds this might not be achievable. 10-12 µm is a compromise to obtain high-quality sections that still allow for IR transmission through most IR regions of interest. Immediately prior to the transfer of a section to a CaF_2_ window, bring the window into close proximity with a deionizer for electrostatic discharge (Haug Biel Deionizer, Mettler Toledo, Mississauga, ON). This prevents unintended movement of sections during collection, and may only be necessary in dry laboratory conditions.
– To transfer a section onto a CaF_2_ window, bring the window into the cryostat (wearing gloves and holding the edges only), and position it directly above the section. The section should jump onto the window and dry rapidly. A pre-cooled paintbrush can be used to facilitate transfer. If transfer results in folding or wrinkles in the section, it can be removed with ultrapure water and isopropanol or acetone.
– Store window in a sterile mini petri dish, section side up, at room temperature. Avoid exposure to light to prevent degradation. Note, for IR, samples must be dry prior to analysis to avoid water interference in the spectra and to prevent movement during the measurement period.

- For BioXAS and SXRMB, section at a thickness of 20 to 80 µm and collect the section on Kapton^®^ or Carbon tape, respectively.

– Section thickness should be decided based on the sample and resolution required. Thinner samples are better for resolution but increase dwell time (requiring longer measurements). A rough guide is to section 1 - 2 cell layers. For pea, sections collected were 40 µm.
– To collect a section on Kapton^®^ or Carbon tape, ensure the block face is trimmed and sections of optimal thickness can be attained. Cut a piece of Kapton^®^/Carbon tape long enough to span the mini petri dish the section will be stored in. Place the adhesive side directly on the block face (with the long edge of the tape running parallel to the cutting plane, perpendicular to the knife edge) and use a pre-cooled cryostat roller to ensure thorough contact between the tape and sample block face. Proceed with section cutting, gently holding the tape along the bottom edge of the block face, to allow contact between the block and knife edge.
– For storage, suspend the section in the middle of a sterile petri dish by connecting the outer edges of the Kapton^®^/Carbon tape with the dish and store at room temperature. In some instances, such as with mobile elements, freeze drying of samples may be required. Additionally, some samples may be stored frozen as, at some beamlines, cryo-measurements with hydrated tissue are possible. Avoid exposure to light to prevent degradation due to oxidation.

When cross-beamline comparisons between regions of interest within a sample will be made, interpretation can be facilitated by selecting cryo-sections in close proximity. If collecting serial cryo-sections, sections should be interspersed rather than collecting for each beamline separately. For example, after collecting a 10 µm section on CaF_2_ window, shift the cryostat to 40 µm step size, cut one section to shift mechanism to new thickness, then collect subsequent 40 µm section on Kapton^®^ tape. Note: for small features such as a globular-stage embryo, the number of serial sections possible is finite.

### 2.5 Experimental parameters for synchrotron radiation-based micro-computed tomography (SR-µCT at BMIT-BM)

At the CLS, two beamlines for SR-µCT analyses are available at BMIT (https://bmit.lightsource.ca/) (Gasilov et al., 2024). The low energy bending magnet (BM) uses 12.6 – 40 keV hard X-rays and the higher energy insertion device (ID) beamline uses 28 – 140 keV. SR-µCT of seeds in this study used the BMIT-BM beamline.

- Detectors. There are multiple detector set-ups available which include both monochromic and white beam at the BMIT beamline at the CLS. These set-ups consist of a scintillator, objective lens and eye piece (0.9x) lens coupled with a camera. Using various magnifications and/or camera combinations, a variety of pixel sizes and fields of view (FOV) can be achieved (for more information see Gasilov et al. 2024). Available objectives are 2X, 5X, 7.5X, 10X and 20X. Objective changes require experienced beamline scientists and time for re-alignment. It is recommended to consult with experienced synchrotron scientists to tailor the imaging set-up to each experiment.
- Filters. When using a filtered white beam, a broad band polychromatic beam is produced. The correct filter combination for collecting SR-µCT scans of live plant materials will limit radiation damage. There are a number of filters and thicknesses available at the BMIT-BM beamline, including aluminum, glassy carbon, silver, molybdenum, copper, and tin. See **Supplementary Table 1** for a list of filters.
- Stage holder. Place the sample on a goniometer head and secure to the beamline stage holder. A goniometer (Goniometer head 1005, Huber Diffraktionstechnik GmbH & Co. KG, Rimsting, Germany) allows for control of sample orientation in the x, x’, y, y’ and z axis to ensure optimal position adjustment for centering the sample within the field of view limitation of the beam. Note that a variety of custom holders are available.
- Data collection. Samples are rotated within the beam over 180° for conventional µCT for 500 - 3000 projections and over 360° for half-acquisition µCT with 1000 - 6000 projections. The range of the vertical stage is 30 - 90 mm depending on the setup. Multiple vertical views can be stitched together in post-processing. The time to scan each vertical view is dependent on the exposure time and the number of projections; as an example, each vertical view for pea took ~5 minutes. Details for all SR-µCT scans are shown in **Table 1**.

**Table 1:** SR-µCT imaging specifications.

	Watermelon	Canola	Pea	Root
Image capture				
Detector	PCO Edge 5.5	PCO Edge 5.5	PCO Edge 5.5	PCO 4000
Objective	Optic Peter 2x	Optic Peter 10x	Optic Peter 5x	Hamamastsu AA60x
Effective magnification	1.8x	9x	4.5x	1x
Scintillator	LuAg: Ce 200 µm	LSO: Tb 10 µm	LuAg: Ce 50 µm	LuAg: Ce 200 µm
Pixel size	3.61 µm	0.72 µm	1.44 µm	9 µm
Sample to detector	0.05 m	0.03 m	0.05 m	0.4 m
White beam	1.000 aluminum filter (mm)	1.000 aluminum filter	0.800 mm Al + 0.100 silver filter (mm)	–
Beam energy	20 keV	20 keV	25.5 keV	41 keV
Mode	conventional	conventional	conventional	half-acquisition*^1^*
Projections	3000 images	3000 images	2500 images	3000 images
Flats	10 images	20 images	50 images	20 images
Darks	10 images	20 images	20 images	20 images
Reconstruction				
Phase	yes	yes	yes	yes
Delta/Beta	300	150	150	400
Spot removal	Yes	Yes	Yes	No
Prominence of spot	10000 counts	1000 counts	5000 counts	–
Spot blur	7 pixels	3 pixels	2 pixels	–
Ring removal	Yes	Yes	Yes	Yes
Fourier-transform	2D	2D	2D	2D
Sigma horizontal	60	60	13	90
Sigma vertical	2	2	2	2
Clipped histogram	No	Yes (16-bit)	Yes (16-bit)	No
Min value	–	4.56E-15	-5.42E-04	NA
Max value	–	1.06E-14	1.46E-04	NA
Detector cropped	yes	yes	no	yes
x (width)	2560	2560	2560	4000
y (height)	1000	1000	2160	888
Field of View				
FOV	2560x2560x1000 pixels	2560x2560x1000 (pixels)	2560x2560x2160 pixels	4000x4000x888 pixels
FOV	9241 x 9241 x 3610 µm	1843 x 1843 x 1555 µm	3816 x 3816 x 3100 µm	36000 x 36000 x 8000 µm

*^1^* Half-acquisition mode is available for larger samples that do not fit within the horizontal field of view. This mode increases the field of view to approximately double that of conventional mode by offsetting the sample to the right or left of the detector and collecting projections over 360˚. Images 180˚ apart are then stitched together to increase the horizontal field of view.

### 2.6 Experimental parameters for Mid-IR spectroscopy (Mid-IR beamline)

Multiple approaches, with or without synchrotron light, are available for the detection of biochemical compounds using Mid-IR spectroscopy depending on the resolution required. Access to both synchrotron IR and offline imaging instrumentation is provided at the Mid-IR beamline at the CLS. When larger samples are scanned, or high resolution with low noise spectra is not required, a globar source with an FPA detector is sufficient (offline). Synchrotron IR (SIR) mapping is used to optimize IR spectra quality, and may be used for acquisitions at the cellular level.

#### 2.6.1 Chemical imaging analysis (developing pea seeds, canola and wheat)

IR imaging maps were obtained at the Mid-Infrared beamline (https://www.lightsource.ca/facilities/beamlines/cls/beamlines/mid-ir.php) at the Canadian Light Source. Offline IR images were acquired at the Agilent end station, equipped with an Agilent FTIR microscope and spectrometer (Agilent Technologies Cary 620 FTIR microscope with 128x128 pixel Focal Plane Array detector, 25x objective and 670 spectrometer, Santa Clara, CA). The spectra were collected in transmission mode in the Mid-IR range of 3900 - 900 cm^-1^ with 16 coadded scans and a spectral resolution of 4 cm^-1^. For the parameters used to scan pea sections, each mm^2^ of tissue area took approximately 13 minutes. The SIR images were acquired using the Bruker end station with a Bruker Vertex70v spectrometer (Bruker Optics Inc, Billerica, MA) with a Hyperion 3000 microscope (MCT-detector) equipped with a 36x objective and 0.3 circular objective in transmission mode in the Mid-IR range of 4000 - 900 cm^-1^ with 8 coadded scans and a spectral resolution of 4 cm^-1^ (**Supplementary Table 2**).

For offline and SIR measurements, section(s) on IR transparent windows were secured to a sample stage. A visible microscope image and IR background were acquired before sample measurement. For the offline Agilent end station, individual IR image tiles were acquired by the FPA detector and stitched together into a mosaic image by the instrument software (see Section 2.11.2 for data analysis). For the SIR Bruker end station, seed IR images were collected with point-by-point mapping the SIR beam spot over the area of interest and collecting an IR spectra at each pixel.

### 2.7 Experimental parameters for XRF-imaging (BioXAS imaging and SXRMB)

There are different beamlines available for XRF imaging, which employ hard X-rays (BioXAS-Imaging) or soft X-rays (SXRMB) to detect different elements. SGM (250 - 2000 eV) and VESPERS (6 - 30 keV) beamlines at the CLS provide XRF imaging with additional energy ranges and capabilities. At BioXAS Imaging, there are macro and micro imaging modes provide unique resolution capabilities. It is important to select dwell times that ensure adequate detection for the element(s) of interest. Prior to initiating an experiment, discussion with experienced beamline staff is recommended to address desired outcomes. See **Supplementary Table 3** for specifications of data collection across all samples.

#### 2.7.1 XRF elemental imaging at BioXAS-imaging

XRF-imaging was performed at the BioXAS-Imaging beamline (https://www.lightsource.ca/facilities/beamlines/cls/beamlines/bioxas-imaging.php#Techniques) at the Canadian Light Source. Here, XRF elemental imaging at BioXAS-Imaging was used to investigate elemental spatial distributions in cryo-sectioned immature pea seed, mature canola seed and mature wheat grain. BioXAS-Imaging is uniquely suited to accessing biologically important trace elements such as Cl, Ca, Zn, Fe, and K based on its X-ray energy range of 5 to 20 keV. At the beamline, high brilliance X-ray light is generated by an in-vacuum undulator insertion device. The primary optics of the beamline consist of a collimating mirror, a fixed-exit double crystal monochromator, and a post-monochromator vertically focusing mirror. For macro-mode imaging, the spot size of the incident X-ray beam onto the sample is shaped by a 20 µm tungsten aperture (40 µm for coarse scans). For micro-mode imaging, a set of Kirkpatrick-Baez micro-focusing mirrors (K-B-mirror) focus the beam to a spot size of 5 x 5 µm. Macro- and micro-mode measurements were collected with incident beam energy set at 13.45 keV (pea and wheat) or 15 keV (canola) and sample positioned at 45° to reduce scattering signal. The XRF signal for XRF-imaging and µ-XAS was collected by a Vortex-ME4 silicon drift X-ray detector for macro-mode and Vortex-ME3 silicon drift X-ray detector for micro-mode (Hitachi High Technologies Science American, Inc., Chatsworth, CA).

For sample measurements, seed sections on Kapton^®^ tape were affixed to the sample plate on the imaging stage with sample side facing the incident beam. A coarse scan was collected for the sample plate to acquire an overview of all samples (macro-mode: 10 msec dwell time and 0.2 mm pixel size; micro-mode: 25 msec dwell time and pixel size to 50 microns). Single point XRF spectra at different dwell times on the sample were collected for optimization of dwell time required to obtain adequate counts for the elements of interest and reduce the total time required to scan the samples. Using the coarse scan, regions of interest were selected and cued for high-resolution measurement (pea: macro-mode 20 µm pixel with 75 msec dwell time; wheat: micro-mode 5 µm pixel with 100 msec dwell time; canola: micro-mode 5 µm with 200 msec dwell time). For the parameters used to scan pea sections, each mm^2^ of tissue area took approximately 3.5 minutes; scan time can be calculated based on dwell time and pixel size.

#### 2.7.2 XRF elemental imaging at SXRMB

XRF-imaging at the Microprobe end station of the SXRMB beamline (1.7 – 10 keV) (https://www.lightsource.ca/facilities/beamlines/cls/beamlines/bioxas-imaging.php#Techniques) at the Canadian Light Source, was used to investigate phytate localization in mature canola seed. SXRMB is uniquely suited to access biologically important elements from Si - Ca due to its optimization to the tender/soft X-ray energy range of 1.7 to 5 keV (Xiao et al., 2017). The X-ray light source is a bending magnet with primary optics consisting of a collimating mirror, double crystal monochromator and toroidal mirror. The microprobe end station slits and K-B-mirror further shape the incident monochromatic beam at 4 keV into a 10 x 10 µm spot. XRF signal was measured by a seven-element silicon drift detector.

For sample measurements, the canola seed section on Kapton^®^ tape was affixed to a Cu sample plate with double sided carbon tape and placed within the vacuum chamber and pumped down. Note, that in this study, the section was originally collected on Kapton^®^ tape, requiring it to be affixed to carbon tape for imaging. The sample holder was placed within the vacuum chamber of the beamline end station and pumped down. A coarse scan of all of the samples on the plate (10 µm beam size, 0.5 s dwell time and pixel size to 50 µm) was collected to show general elemental distribution and highlight areas of interest for high resolution scans. These parameters result in a scan time per 1 mm^2^ of section of approximately 3.5 minutes. High resolution scans at selected areas were acquired with 10 µm resolution. The resulting XRF signal of P was calculated over the P K α1 range from 1952 - 2075.4 eV.

### 2.8 Experimental parameters for µ-XAS collection of XANES spectra (BioXAS imaging and SXRMB)

#### 2.8.1 Select the spot(s) of interest for µ-XAS

The XRF elemental map collected (Section 2.7.1 for BioXAS-Imaging and Section 2.7.2 for SXRMB imaging) can be used for µ-XAS measurement to identify area(s) of interest and the exact coordinate(s) with respect to the sample stage. For the pea zinc XANES spectra, a coarse scan macro-mode XRF map was collected at BioXAS at 10 keV with 40 µm pixel size. Once a region of interest was selected, a µ-XAS measurement at the coordinates was setup to collect XANES data. For the pea seed Zn K-edge (9659 eV), XANES spectra were collected from 9558.6 – 9795.3 eV. For µ-XAS measurement at SXRMB of P in canola, regions of interest can be set based on fine scans and P K-edge XANES collected from 2115 – 2210 eV. Replicate XANES measurements are strongly recommended at the same spot and/or nearby spot(s) within the same tissue. In the case of pea seed, a minimum of 4 XANES spectra at neighbouring spots were collected for each area of interest. Scan times are dependent on the required dwell time, the number of energies collected and the number of spots measured.

### 2.9 Experimental parameters for Scanning transmission x-ray microscopy

The X-ray imaging and spectroscopy were performed using the spectromicroscopy (SM, 10ID-1) beamline (https://www.lightsource.ca//facilities/beamlines/cls/beamlines/sm.php#top) at the Canadian Light Source (Kaznatcheev et al., 2007), to investigate subcellular P distributions in mature canola seed embryonic cotyledons. The raw transmitted X-ray flux signals were converted to optical densities (OD) (i.e., absorbance) using an incident flux signal measured at regions devoid of sample material. P was mapped in canola sections using the P K-edge. Specifically, off-resonance (2143 eV) and on-resonance (2155 eV) OD images were collected for the same area at a spatial resolution of 50 nm, and the difference of the images (i.e., subtraction) resulted in the P map (Dynes et al., 2015) (See **Supplementary Table 3**). The P map was then overlaid with the off-resonance map. The off-resonance map represents the non-P components, mainly the organic components. The P K-edge spectra were obtained by collecting a sequence of images (i.e., stack) at specific energies for AlPO_4_ and selected canola seeds. The energy scale was calibrated using AlPO_4_ (2154.5 eV) (Tofoni et al., 2023). Note that when the data was collected, a new monochromator was being commissioned on the SM beamline, and the energy scale was not reproducible, so the energy scale of the canola seeds is not expected to be accurate. Analyses were performed with aXis2000 (http://unicorn.mcmaster.ca/aXis2000.html; Hitchcock, 2023).

### 2.10 Complementary methods

ESEM images were collected using a Thermo Quattro S SEM at the Electron Imaging and Microanalysis Lab at the Canadian Light Source. Backscattered electron images were collected at ESEM pressure of 150 Pa with beam energy of 5 keV.

Oil and protein contents of NAM13 and NAM45 seeds were determined using NIR analysis (NIRS system model 6500, FOSS NIRSystems, Silver Springs, MD).

Phytate content of oil-free seed residue was determined by Phytate/Phosphorus assay (Megazyme K-PHYT, Megazyme, Wicklow, Ireland).

To remove oil from canola seeds, seeds were split in half using a razor blade. One half of the seed was soaked in 10 mL reagent grade mixed hexanes in a glass vial for 1 hour. The seed was removed, air dried, then placed in a clean vial.

### 2.11 Data analysis

#### 2.11.1 SR-µCT image processing (BMIT-BM)

Image sequences were reconstructed and vertically stitched using ufo-kit tofu software (GitHub - ufo-kit/tofu: Helper scripts for tomographic reconstruction using the ufo-core framework (https://github.com/ufo-kit/tofu) (Faragó et al., 2022). During reconstruction of the projected images, flat field correction (FFC) was performed using the acquired flat and dark signal images. Paganin TIE (transport-of-intensity equation) phase retrieval, a non-interferometric non-iterative method of deterministically solving the TIE using the intensity distribution at the in-focus plane and the axial intensity derivative (Zuo et al., 2020) was enabled. This increases the signal to noise ratio and the image contrast, especially with respect to water and plant tissue within the sample. Large spots were removed using predetermined thresholds. A Fourier-transform based filter was used to remove ring artifacts resulting from sample rotation. Vertical stitching was performed based on the image row overlap between adjacent vertical scans. The resulting 32-bit image sequence was clipped and converted to 16-bit where required. The images were then cropped as required to minimize background information using ImageJ software (https://imagej.net/ij/) (Schneider et al., 2012). The time required for image processing is dependent on the available computational resources but each sample can take a minimum of 1-2 hours to reconstruct and stitch with a high performance computer (current computer specifications comprised a Precision 7820 Tower with two Intel Xeon Silver 4215R processors, two RTX A8000, 48GB graphics cards and 512 GB RAM). Following the reconstruction, the target image sequence for segmentation with Avizo should be less than 20 GB. Image sequences were loaded into Avizo software (Thermo Fisher Scientific, Waltham, MA) for segmentation. Every ~40th slice was segmented and a smart interpolation of the image sequence segmentation was performed using Biomedisa (https://biomedisa.info/) (Lösel et al., 2020). Biomedisa results were returned to Avizo for the final segmentation, volume rendering and analysis. Reconstruction parameters for all samples are shown in **Table 1**. For additional methodology descriptions see Willick et al., 2020.

#### 2.11.2 MidIR data processing (MidIR beamline)

The data output file was loaded into Quasar software (https://quasar.codes/) (Toplak et al., 2017, Toplak et al., 2021). The spectra were pre-processed to correct for atmospheric gas (CO_2_/H_2_O) using default parameters. A rubber band baseline correction was performed. For visualization of peak areas, baseline integrals from peaks of interest were calculated; specifically, 2819 - 2994 cm^-1^, (lipid-rich, CH_2_ and CH_3_), 1700 - 1770 cm^-1^ (C=O stretch; lipid), 1600 - 1700 cm^-1^ (amide I), 1490 - 1580 cm^-1^ (amide II), 1200 - 1490 cm^-1^ (structural carbohydrates), and 900 - 1200 cm^-1^ (carbohydrate). Empty pixels were removed from the spectra based on thresholds in the fingerprint region (1800 cm^-1^ - 900 cm^-1^). For the PCA and K-means cluster analysis shown, data was further processed by removing empty pixels through thresholding, limited to the fingerprint region (1800 cm^-1^ - 900 cm^-1^), baseline corrected and vector normalized. All data was exported from Quasar for subsequent visualization in R (ggplot2) or Python (Matplotlib).

#### 2.11.3 XRF data processing (BioXAS-imaging and SXRMB)

For XRF maps (generated at BioXAS-Imaging), the data file was loaded into PyMca software (PyMca 5.9.2 — https://www.silx.org/doc/PyMca/dev/index.html; Solé et al., 2007). An energy calibration was generated using the Fe K α _1_ and Ca K α _1_ peaks. A fit configuration file containing e.g. fit, background, beam and elements peak parameters was setup and used for batch fitting of the XRF map. The RGB visualization was generated within PyMca RBG correlator’s matplotlib visualization tool. For the SXRMB XRF maps, the elemental maps were generated from the resulting XRF spectrum based on appropriate energy windows (P K α _1_ 1952 - 2075.4 eV, Ca K α _1_ 3608.14 - 3775.22 eV, Cd L α _1_ 3056.76 – 3210.7 eV) normalized with incident I0.

#### 2.11.4 XANES data processing (BioXAS-imaging and SXRMB)

XANES data were processed using Athena software (https://bruceravel.github.io/demeter/aug/index.html ; Ravel and Newville, 2005). X-ray absorption spectra were calculated by dividing raw fluorescence with incident I0 signal. Pre-edge and post-edge parameters were adjusted to determine the pre-edge and normalization lines respectively. Normalized XANES spectra were calculated by subtraction of the pre-edge line, followed by division by the normalization constant determined by the pre-edge and normalization line. For visualization, data was exported and plotted using Origin Pro Software (OriginLab, Northampton, MA).

## 3 Results

**Figure 2:**
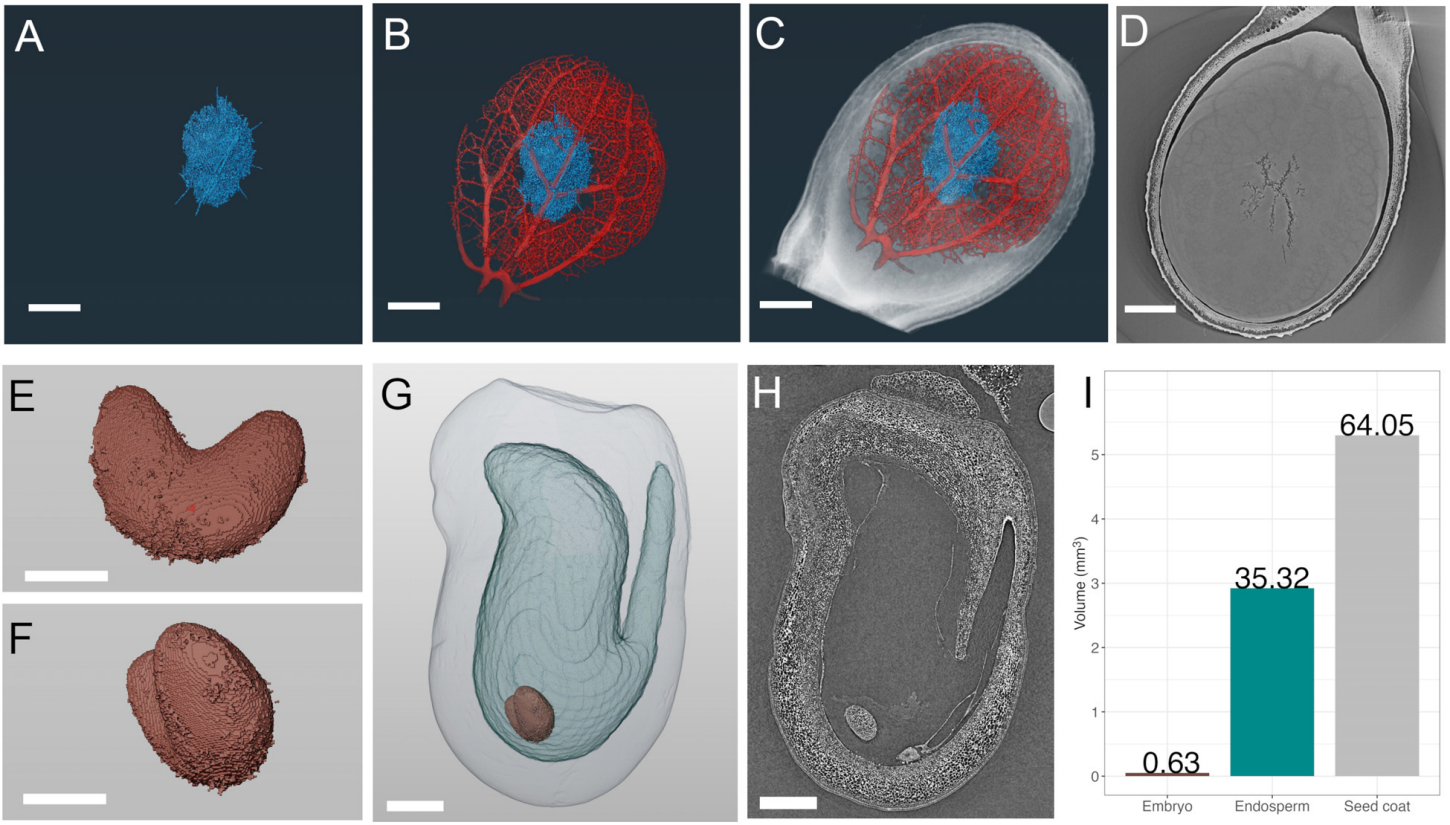
SR-µCT of mature watermelon (*Citrullus lanatus*) and immature pea (*Pisum sativum*) seeds produced at the Canadian Light Source BMIT-BM beamline. SR-µCT from a mature watermelon seed infected with *Acidovorax citrulli* **(A-D)** and a developing pea seed 7 days post flowering **(E-H)**. **(A)** Segmented and volume rendered necrotic path of bacterial fruit blotch infection alone **(A)**, in blue) and overlaid with volume rendered seed vasculature **(B)**, red), and whole watermelon seed **(C, D)** SR-µCT slice through infected watermelon seed, **(E, F)**. Segmented and volume rendered embryo from an early stage pea seed. **(G)** Segmented and volume rendered whole pea seed of seed coat (outermost light grey), endosperm cavity (light blue) and embryo (red) subcompartments. **(H)** SR-µCT slice through a pea seed. **(I)** Percent volume of each subcompartment of the pea seed in **(G)**. Scale bar **(A–D)**, 1000 µm; scale bar **(E, F)**, 250 µm; scale bar **(G, H)**, 500 µm.

**Figure 3:**
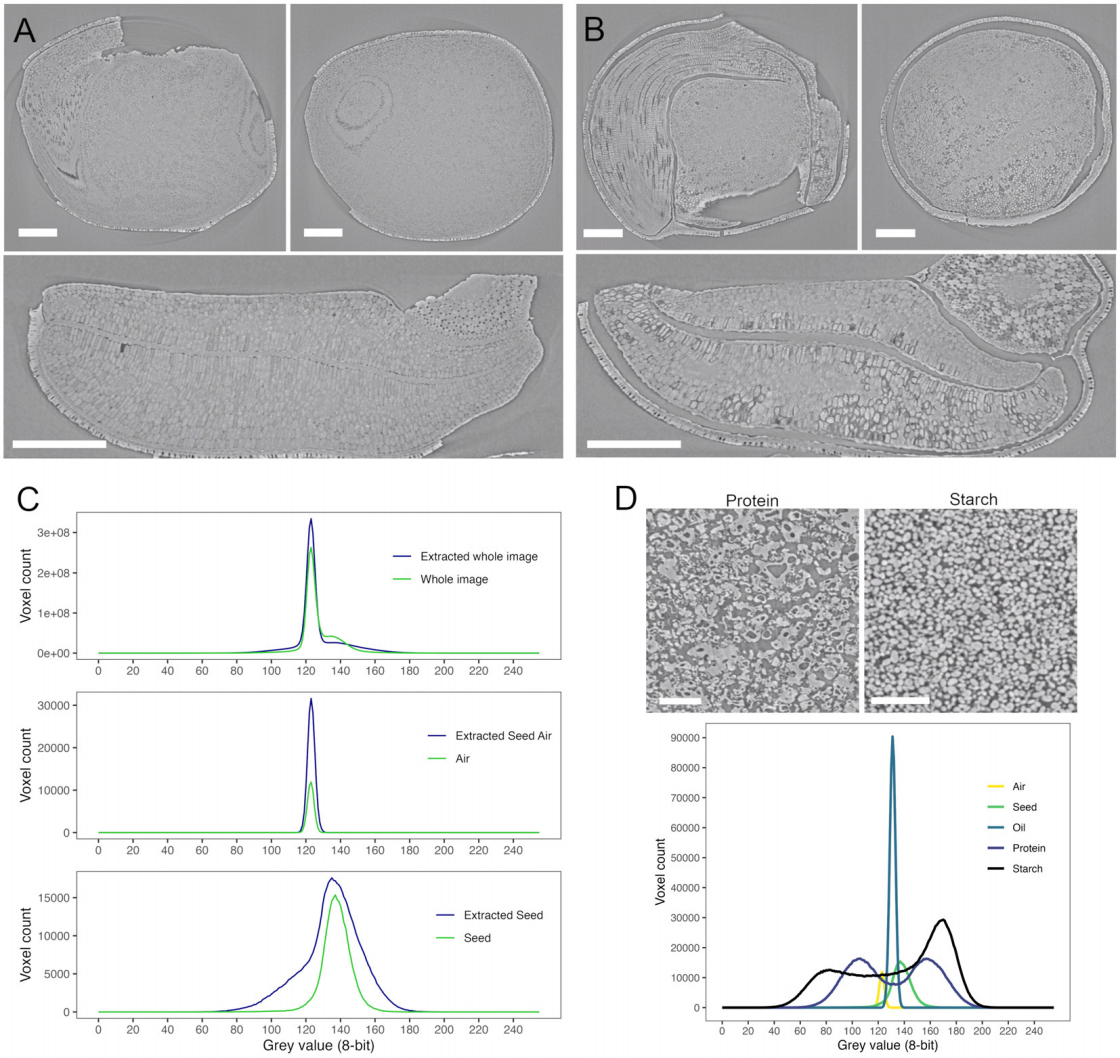
SR-µCT of a mature canola (*Brassica napus*) seed, produced at the Canadian Light Source BMIT-BM beamline. An SR-µCT slice is shown before **(A)** and after **(B)** extraction with hexane. Histograms of the 8-bit grey value for the seed, air and total image are shown in panel **(C)** SR-µCT of isolated protein and starch (panel **(D)** show distinct morphological differences as well as distinct grey values as seen in the histogram below. Scale bar **(A, B)** 200 µm; scale bar **(D)**, 100 µm.

**Figure 4:**
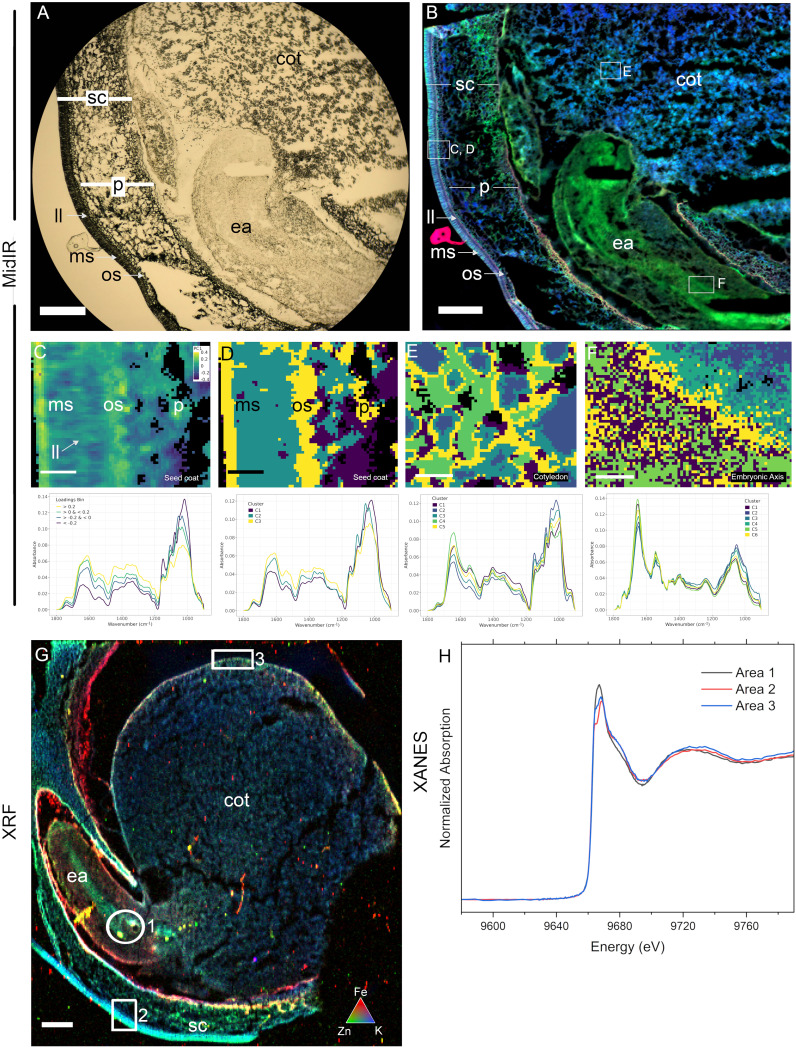
Cross-beamline semi-correlative analysis of nutrient distributions in a single immature pea (*Pisum sativum*) seed, produced at the Canadian Light Source MidIR spectroscopy and BioXAS-Imaging beamlines. Brightfield/transmitted light view of a cryosectioned pea seed showing the visual region scanned **(A)** and the resulting reconstructed MidIR image showing lipid (red), protein (green) and carbohydrate (blue) distributions **(B)**. Selected regions in **(B)** including in the seed coat **(C, D)**, cotyledon **(E)**, and embryonic axis **(F)** were assessed using principal component analysis **(C)** and K-means clustering **(D-F)**. An adjacent section was used for XRF imaging and an overlay of iron (red), zinc (green) and potassium (blue) distributions are shown **(G)**. Zinc speciation differences were investigated in thee selected regions in G using XANES **(H)**; areas 1, 2, and 3). Scale bar **(A, B, G)**, 500 µm; scale bar **(C–F)**, 50 µm. sc, seed coat; p, parenchyma; ll, light line; ms, macrosclereids; os, osteosclereids; ea, embryonic axis; cot, cotyledon.

**Figure 5:**
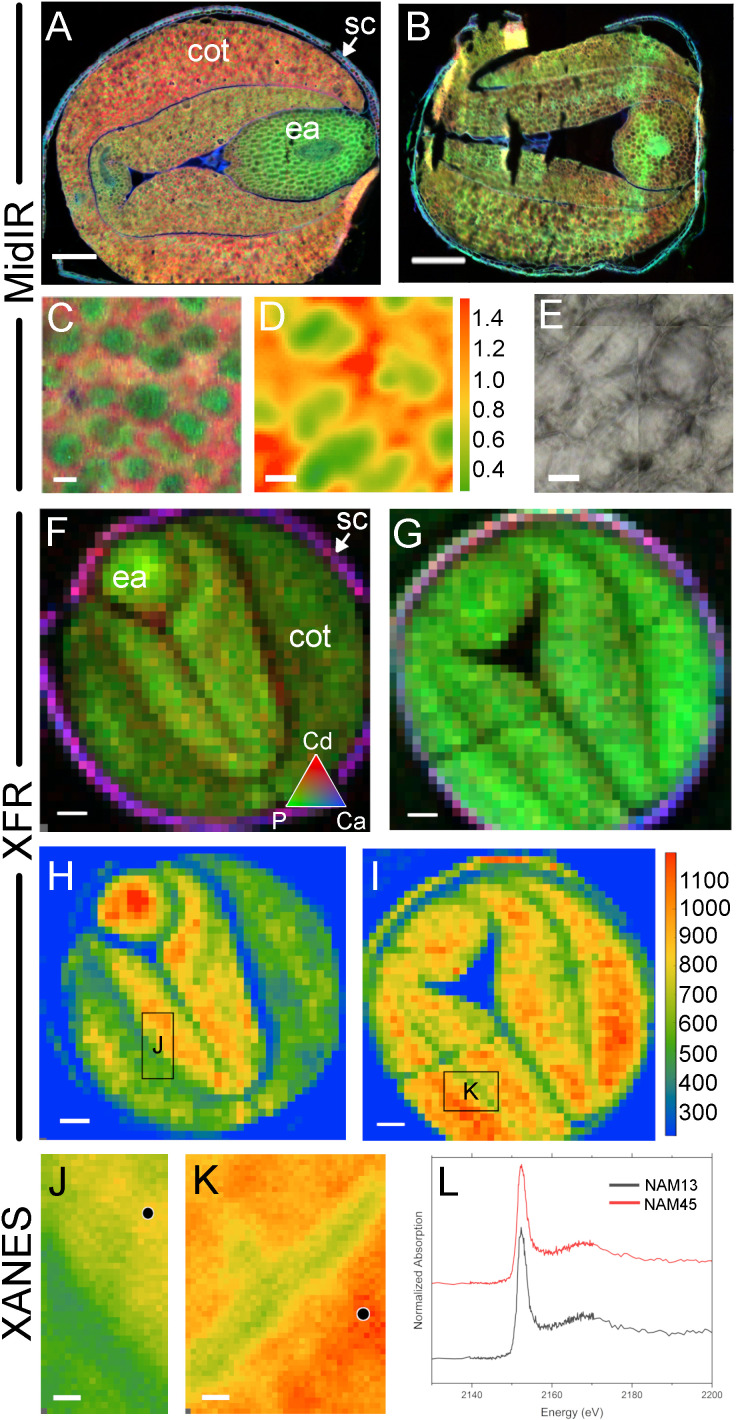
Cross-beamline analysis of nutrient distributions in high and low phytate mature canola (*Brassica napus*) seed, produced at the Canadian Light Source MidIR spectroscopy and SXRMB beamlines. Cryosectioned mature seed in a high level phytate genotype, NAM13 **(A, C-F, H, J)** and a low level phytate genotype, NAM45, **(B, G, I, K)**. MidIR reconstructed images of canola seed lipid (red), protein (green) and carbohydrate (blue) macronutrient distributions in NAM13 **(A)** and NAM45 **(B)**. High magnification images shows the tissue distribution of protein bodies (green) surrounded by lipid (red) with conventional IR source **(C)** and the lipid to protein rations with a synchrotron IR source **(D)** and corresponding visual image **(E)**. These same seeds were used to generate XRF images at the SXRMB to reveal cadmium (red), phosphate (green) and calcium (blue) distributions **(F, G)**, and the specific distribution of phosphate **(H, I)**. Regions of interest in the high phytate **(J)** and low phytate **(K)** genotype were scanned at higher resolution and black dots indicate specific regions for subsequent XANES **(L)**. Scale bar **(A, B, F-I)**, 200 µm; scale bar **(C-E)**, 10 µm.

**Figure 6:**
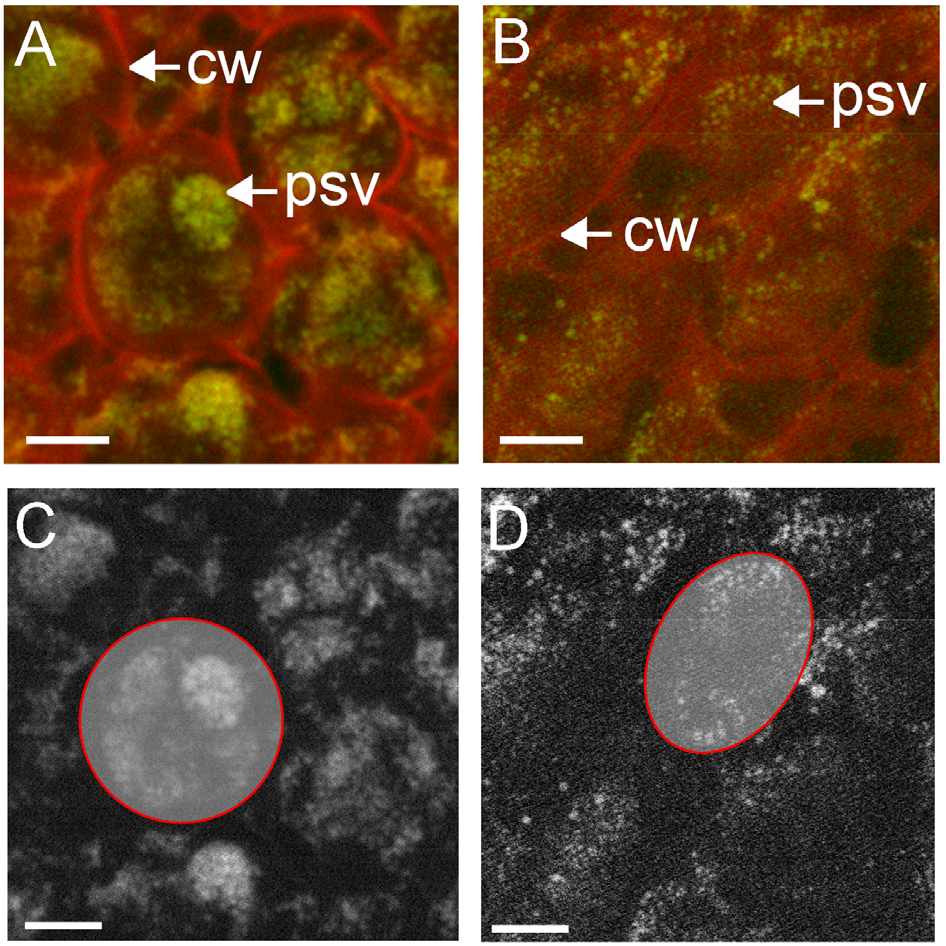
Subcellular distribution of phosphorus in cotyledon cells of high phytate **(A, C)** and low phytate **(B, D)** genotypes, measure with STXM at SM. Phosphorus (green) was mapped using the P K-edge, with on-resonance (2155 eV) and off-resonance (2143 eV) and shown against off-resonance optical density (red) **(A, B)**. Panels **(C, D)** are greyscale representations of phosphorus in **(A, B)**, respectively, with individual cells traced in red for semi-quantitative analysis of phosphorus content. Scale bar 10 µm. cw, cell wall; psv, protein storage vacuole.

**Figure 7:**
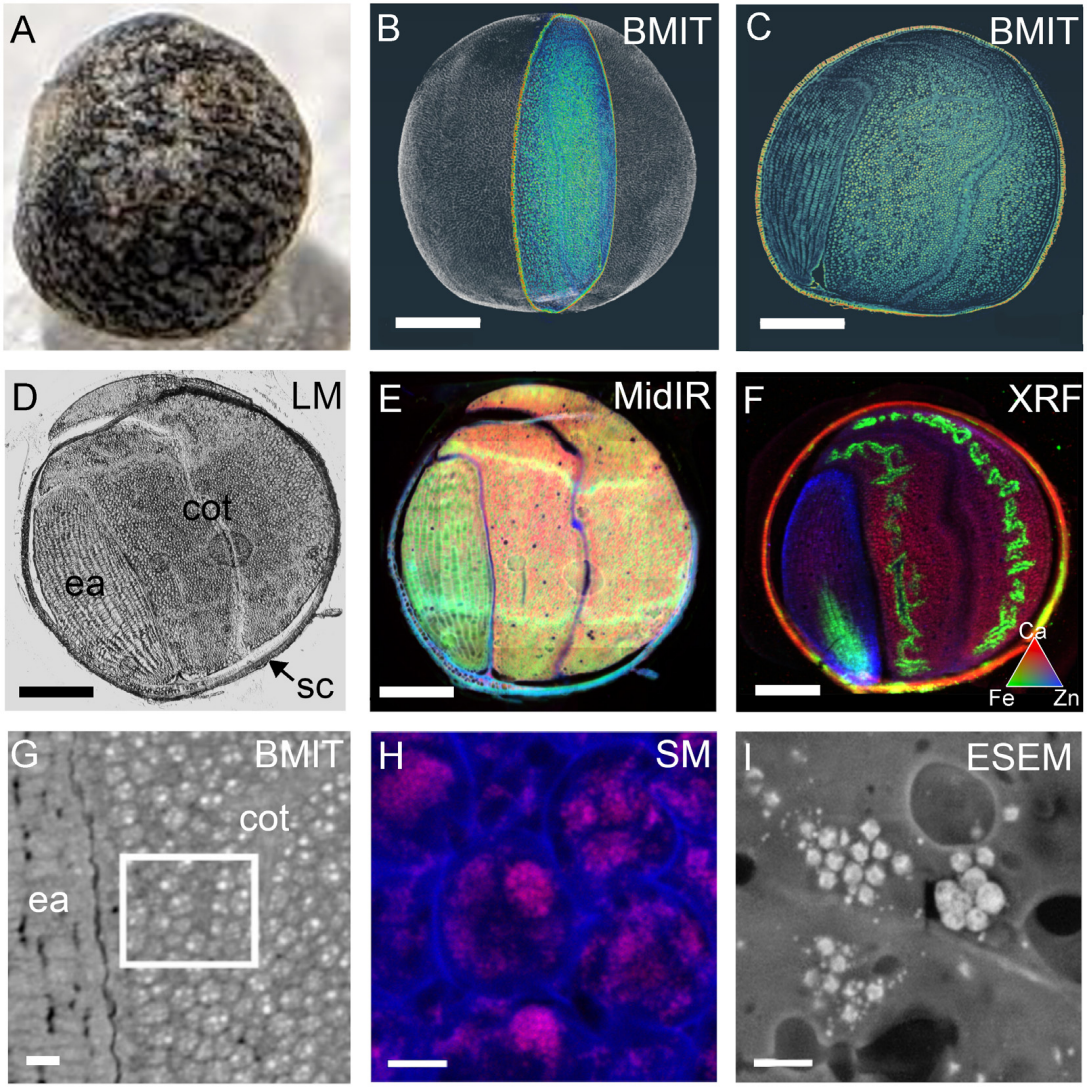
Cross-beamline correlative analysis of structure and nutrient distributions within a single mature canola (*Brassica napus*) seed, at the Canadian Light Source MidIR spectroscopy, BioXAS-Imaging, SM and Electron Imaging and Microanalysis Lab or EIML beamlines. A photograph of the mature, intact canola seed **(A)** and the 3D rendering following SR-µCT with a single section highlighted **(B)**. The SR-µCT single slice view **(C)** of the highlighted section in **(B)** corresponds to the same slice imaged by light microscopy **(D)**, MidIR spectroscopy **(E)**, and XRF mapping **(F)** following cryosectioning. MidIR spectroscopy **(E)** shows the distribution of lipid (red), protein (green) and carbohydrate (blue) and the XRF mapping **(F)** shows calcium (red), iron (green) and zinc (blue) distributions. A higher resolution slice showing cellular detail by SR-µCT **(G)**, white box) can be used to inform the locale for phosphorus mapping by SM **(H)**. Environmental scanning electron micrograph (ESEM) **(I)** shows protein storage vacuoles and globoids. Scale bar **(B, F)**, 500 µm; scale bar **(G)**, 50 µm; scale bar **(H)** 10 µm; scale bar **(I)**, 2 µm. sc, seed coat; ea, embryonic axis; cot, cotyledon.

**Figure 8:**
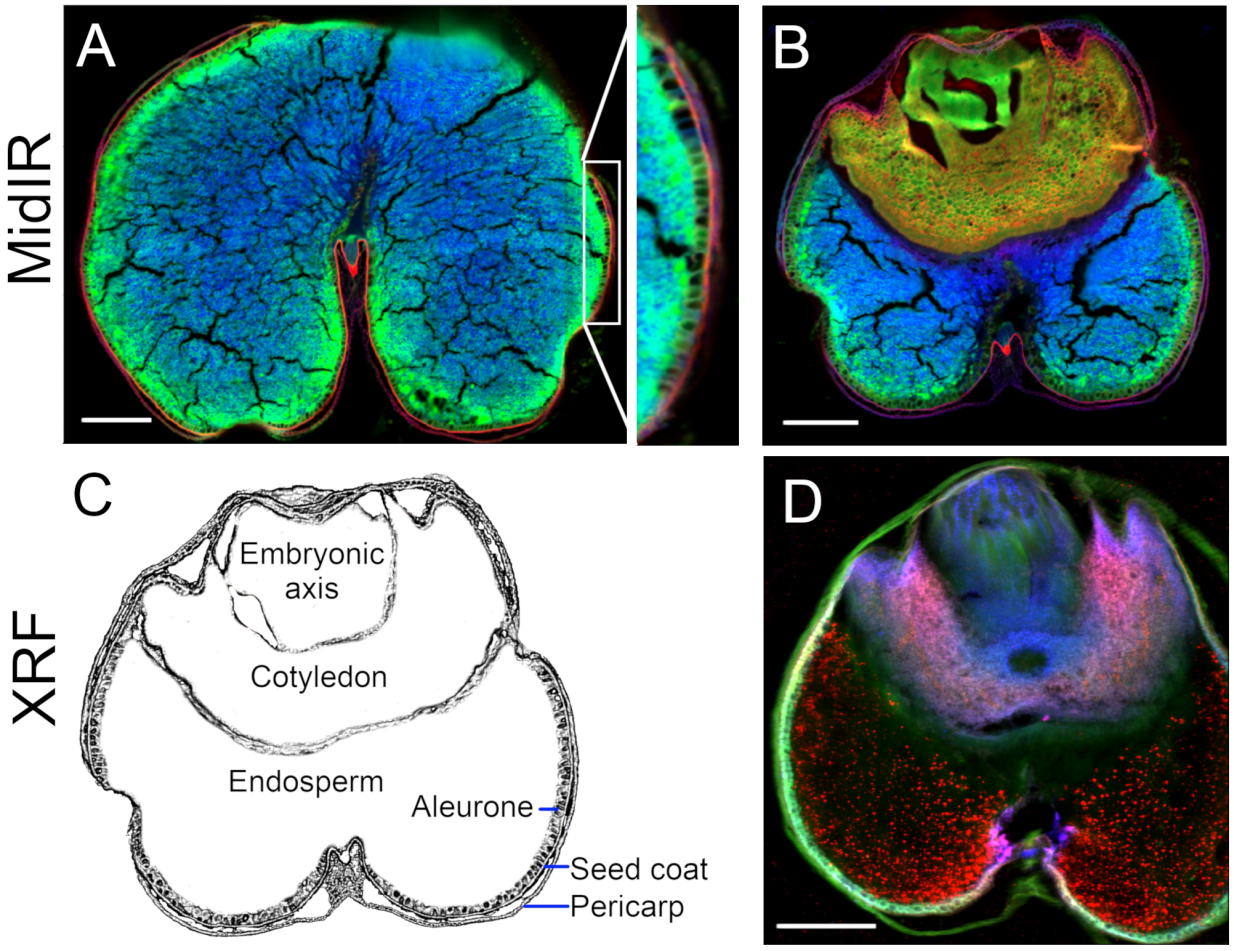
Cross-beamline analysis of nutrient distributions in mature wheat (*Triticum durum*) grain, produced at the Canadian Light Source MidIR spectroscopy and BioXAS-Imaging beamlines. MidIR spectroscopy **(A, B)** shows lipid (red), protein (green) and carbohydrate-enriched (blue) distribution patterns in mature wheat seeds in the endosperm **(A)** and embryo **(B)** regions of the seed. A simplified rendering of a wheat grain section with labels is provided for reference **(C)**. XRF imaging shows the distribution of calcium (red), potassium (green) and manganese (blue) through the embryo, endosperm and seed coat **(D)**. Scale bar 500 µm.

To support the adoption of synchrotron-informed research in plant sciences, we present methods for "preparing an array of crop seeds for analysis by a wide range of electromagnetic radiation energies. An overview of method pipelines is illustrated in **Figure 1**, starting with input options for seeds to" accommodate mature, dry materials or developing, fresh seeds (dark green panels). Sample inputs for SR-µCT imaging (at the BMIT-BM beamline) can accommodate whole seeds without the need for sectioning, and benefits from the preparation of dry materials (**Figure 1** yellow panels). Preparation of seed materials for all other beamlines described here involved spatially-resolving chemical compositional data and thus, used hydrated, frozen materials that were cryo-sectioned (and not chemically fixed; **Figure 1** light green boxes). Note that XRF imaging on hydrated, non-sectioned, thin samples is a viable option depending on the element of interest if complementary multimodal imaging is not required. Utilizing the range of energies available at the CLS, from hard X-rays (5 - 40 keV; **Figure 1** grey boxes), to soft X-rays (130 eV - 10 keV; **Figure 1** blue boxes), and IR light (with or without SR) provides diverse opportunities for making biological discoveries. We focus on the structural information provided by SR-µCT (BMIT-BM) for revealing internal seed anatomy, and the chemical mapping tools for localizing chemistries across seed subcompartments, provided by hard X-ray synchrotron imaging technologies (BioXAS-Imaging beamline) and soft X-ray modalities (SXRMB and SM beamlines), as well as MidIR spectroscopy. Potential data outputs from each of these imaging techniques are summarized by beamline (**Figure 1** white boxes), including the category of molecules that can be probed and the spatial resolutions that can be achieved. Data presented here features case study examples, using seeds from *Citrullus* sp. (watermelon), *Brassica* sp. (canola), *Pisum sativum* (pea), and *Triticum durum* (wheat) seeds (see summary lists at the bottom of **Figure 1**, **Figures 2**–**8**, and **Supplementary Table 4** for a list of Figures, samples and methods), and showcase the combined power of cross-beamline synchrotron imaging for deciphering complex living systems such as seeds.

Mature seeds are an ideal material for SR-µCT as they are easy to manipulate, require no fixation or pre-processing (**Figure 1**) and are dry at maturity, making them subject to minimal motion artifacts during scans. Further, SR-µCT imaging provides non-destructive interrogation of seed traits, after which seeds can remain viable. Case study 1 demonstrates the use of SR-µCT to image watermelon seeds (**Figures 2A–D**). Differential absorption of X-rays relates directly to density, and allowed details of seed anatomy and necrosis due to invading organisms to be distinguished. Pan-through videos of sequential SR-µCT slices through a healthy and a fruit blotch infected seed illustrate this point (**Supplementary Video 1**). Volume renderings following image segmentation isolated internal 3D features of interest including necrotic tissue (**Figure 2A**, blue) resulting from *Acidovorax citrulli*-induced bacterial fruit blotch, and seed vasculature (**Figure 2B**, red). Feature overlays (**Figure 2C**) allowed direct comparison of vasculature and necrotic networks within the seed. The volume rendering was critical to inform the degree of necrosis and the complexity of the vascular network. This was emphasized by a pan-through video of the segmented seed (**Supplementary Video 2**) differentiating the collective network of necrotic tissue from the vasculature and surrounding seed tissue in 3D space (**Supplementary Video 3**). An example of an SR-µCT slice used in image segmentation for 3D rendering is provided (**Figure 2D**).

Computational analysis of SR-µCT data facilitates accurate developmental staging and quantification of internal features following volume rendering as discussed in case study 2 and as shown in **Figures 2E–I**. Individual segmentation of immature pea seed subcompartments, revealed the embryo (**Figure 2E, F**) endosperm cavity (**Figure 2G**), and seed coat (**Figure 2G**) from SR-µCT reconstructed slices (**Figure 2H**). Volumetric analysis showed that the seed coat accounts for 64%, the endosperm cavity for 35% and the embryo for ~1% of the total volume of a seed (**Figure 2I**) at 7 days post flowering. In addition to this quantitative volumetric analysis, precise developmental staging information was readily accessible from 3D reconstructed data, which showed the presence of an early heart embryo. A single slice through a seed viewed by light or transmission electron microscopy (comparable to **Figure 2H**), in this case, could lead to inaccurate classification of the embryo as globular. Large and mature seeds with less permeable seed coats are susceptible to fixative penetration issues (**Supplementary Methods**). If volumetric quantification is not required, seeds can be cut in half and imaged (**Supplementary Figure 1**). This partial seed scan still gives important 3D architectural and developmental information and is useful for correlation with data from matched samples at different beamlines. When the requirement for imaging of plant materials exceeds the size capacity of the BMIT-BM beamline, the higher energies available at the BMIT-ID beamline can be applied. For reference, the complex subterranean root architecture of wheat is shown in **Supplementary Figure 2.** This highlights the capacity for BMIT-ID SR-µCT to isolate living plant features within a complex static matrix such as soil, and demonstrates the ability to investigate larger scale structures. The higher energy BMIT-ID beamline also has the ability to image significantly more dense materials than the BMIT-BM beamline.

SR-µCT is most often used for structural imaging. However, it is possible to glean some compositional information for biological materials using phase retrieval. These scans generate contrast based on differences in attenuation coefficients within the material which relate to the density of the material and the atomic weight of its elements. The application of Paganin propagation-based phase retrieval can increase the contrast in the generated images using differences in refractive indices. This is especially critical in biological samples where materials have similar absorption profiles and contain low atomic number elements. Grey value distribution can be used to differentiate between materials and serve to provide compositional insight. Histograms over single SR-µCT slices through a mature canola seed without extraction (**Figure 3A**) and after hexane extraction of lipids (**Figure 3B**) showed differences across the complete image (**Figure 3C**, top panel) where extraction with hexane sharpened the histogram peak. Hexane extraction increased the air component in the sample (**Figure 3C**, middle panel) and produced differences in the seed histogram (**Figure 3C**, bottom panel). Following extraction, the histogram of the seed component widened to include a larger number of grey values, indicating increased heterogeneity of particle size and/or pore distribution, and the peak of the seed was shifted to the right from that of the air (**Figure 3C**, middle panel) or complete seed (**Figure 3C**, top panel) (peak grey value of 135 versus 123). The increased greyscale range of the extracted seed relates primarily to the increased air volume within the sample. The sample to detector distance and the δ / β ratio (where δ is the phase shift and β is the absorption component of the refractive index) used for phase retrieval during image reconstruction impacts the image quality and the size of the fringe effect and can therefore affect the distribution of relative greyscale values. SR-µCT scans of isolated protein (**Figure 3D**, upper left), starch (**Figure 3D**, upper right) and oil showed distinct morphologies in packing density and particle size. For example, starch has smaller air spaces between granules than protein and this size difference resulted in a shift of approximately 20 grey values for the air peak between samples (**Figure 3D**, lower). These peaks were also shifted from that of bulk air. This demonstrated that air can manifest greater than 20% of the dynamic grey value CT range and irrespective of material, the histogram can inform particle size and amount. The use of hexane extraction resolved the localization of oil versus protein given the histogram peaks overlap. Using these relative greyscale values it is possible, over selected regions within a seed, to gather information regarding localized chemical composition based on phase shifts and comparison of the resulting grey values. An example of this microstructural characterization approach is described by Sivakumar et al., 2024. It should be noted that these changes in grey values are relative changes based on material density, not based on calibration to known standards. Transformation to standard values, as often seen in medical imaging (Hounsfield units), is not practical as air is not a single defined value.

While SR-µCT can support broad classification of chemical components in a sample and their localization patterns, MidIR spectroscopy and XRF imaging are more useful for interrogating macromolecule and element distributions in seeds (case study 2), including through the differentiation steps that form plant embryos. Processing of developing seeds for MidIR and XRF imaging (**Figure 1**) is more involved than for mature seeds. **Figure 4A** shows a visual of the section quality of a 10 µm cryosection for MidIR. The structure and general morphology are intact however, freezing and cryosectioning can create holes in the tissue. The resulting MidIR image (**Figure 4B**) showing lipid (red), protein (green) and carbohydrate (blue) demonstrates the minimal impacts of these sample preparation artifacts on data quality. In the case of pea, developing seeds (13 days post flowering) presented elevated levels of protein in the embryonic axis relative to the carbohydrate-rich cotyledons. The seed coat contained high carbohydrate levels, with protein enriched sub-domains (e.g. at the base of the sclereid layers) and lipid enrichment (particularly apparent in the light line region of the macrosclereid layer).

Generalized overviews of macromolecule distribution provided by MidIR reconstructed images, can be analyzed in regions of interest by PCA (**Figures 4C, B**) and K-means clustering (**Figures 4D, E**) to identify compositional clustering features within the seed coat (**Figures 4C, D**), cotyledon (**Figure 4E**) and embryonic axis (**Figure 4F**). In the seed coat PCA (**Figure 4C**), principal component 1 (PC1), explained 47% of the variation, and showed distinct macromolecule compositions. Binning of the loadings according to the legend breaks followed by extraction and averaging of the spectra values allowed interrogation of the spectra to determine which macromolecules were the primary components at distinct cellular locations. The results of this binning method were in accordance with that of K-means clustering of the fingerprint regions from 900 - 1800 cm^-1^ (**Figure 4D**) where the cuticle and inner layer of the seed coat sclereids (yellow) grouped together in cluster 3. This cluster is consistent with the higher protein content regions shown in the false-colour RGB image (**Figure 4B**) as the average spectra in the cluster analysis (lower panel) showed well defined amide I (1600 - 1700 cm^-1^) and amide II (1490 - 1580 cm^-1^) peaks. Cluster 3 represented elevated structural carbohydrates (1200 - 1490 cm^-1^) and the presence of a lipid component (peak at ~1730 - 1745 cm^-1^) in the predicted macrosclereid light line. Cluster 2 represented a carbohydrate-rich area (see blue in panel B) and the average spectra showed a strong carbohydrate peak (900 - 1200 cm^-1^) with specific peaks likely representing structural carbohydrates such as cellulose (~1110 cm^-1^) and xyloglucans (~1070 cm^-1^). K-means clustering in the cotyledon (**Figure 4E**) showed distinct regions of macromolecule distribution. Vacuole-like structures (blue, cluster 2 inner and cluster 3 outer) were tightly surrounded by a third cluster (yellow, cluster 5). Interrogation of the average spectra across these clusters (**Figure 4E**) supports carbohydrate-rich (900 - 1200 cm^-1^) vacuole-like structures surrounded by high protein regions (cluster 4 and cluster 5) as seen by amide I (1600 - 1700 cm^-1^) and amide II (1490 - 1580 cm^-1^) peaks. The embryonic axis is a protein-rich region according to the false colour RGB image (**Figure 4B**) and K-means cluster analysis corroborates this observation. There are small differences across the region that can be differentiated (**Figure 4F**). However, average spectra (**Figure 4F**) of these clusters show minimal differences and mainly serve to highlight the pronounced amide I (1600 - 1700 cm^-1^) and amide II (1490 - 1580 cm^-1^) peaks in each of the clusters. There are subtle changes in the carbohydrate composition (900 - 1200 cm^-1^) that contribute to cluster assignment.

In addition to macromolecule data for MidIR imaging, multi element data can be obtained from XRF spectroscopy of adjacent sections. A false-coloured reconstructed image depicts Fe (red), Zn (green) and K (blue) distributions in an immature pea seed (**Figure 4G**). Fe was evident in the remnants of endosperm adjacent to the seed coat, while K was more abundant in the seed coat sclereids; a distribution overlapping with that of clusters 2 and 3 from the MidIR analysis (**Figure 4D**). The highest levels of Zn were seen in the inner and outer layers of the seed coat, as well as the central region of the embryonic axis and outer edge of the cotyledons. The distribution of Zn in the embryonic axis overlapped with the region of the greatest protein content, as seen in the MidIR image (**Figure 4B**). Regions with high levels of Zn were selected (1-3) for XANES (**Figure 4G**) and results indicated the presence of different species of Zn across these regions (**Figure 4H**). Calibration standards were not available so conclusive identification of the species requires future investigation.

Case study 3 involved the spatial localization of P in high phytate (NAM13) and low phytate (NAM45) canola lines using multi-beamline SR imaging. Additional information regarding these genotypes is available in **Supplementary Table 5** and in Brown et al., 2024. Phytate analysis demonstrated that NAM13 and NAM45 contain 4.35% and 0.19% phytate, respectively (**Table 2**). These seeds differed in total oil and protein content with the high phytate NAM13 having 13.6% higher oil and 9.1% lower protein than the low phytate NAM45. Using MidIR spectroscopy, the spatial distributions of oil (lipid) and protein across canola seed subcompartments were interrogated. Comparison of RGB (R, lipid; G, protein; B, carbohydrate) images of the high (**Figure 5A**) and low (**Figure 5B**) phytate canola genotypes corroborated the whole seed NIR results. Additionally, MidIR images showed that oil was localized in the cotyledons of the high phytate line with the highest levels in the outer lobes while the low phytate genotype had a more homogeneous distribution of oil. Protein was present in the cotyledons of the high phytate genotype but the highest levels were within the embryonic axis. The low phytate genotype had a relatively homogeneous distribution of protein. Carbohydrate was predominantly seen in the seed coat and lining the outer epidermal edge of the embryo in both genotypes. Since lipid and protein in the cotyledons appeared to differ in their predominant distribution patterns, a high magnification scan of this region (**Figure 5C**) in the high phytate genotype was performed. This showed protein concentrated in distinct regions of the cell, in a lipid-rich matrix. This was supported by SIR and calculation of the lipid to protein ratio (**Figure 5D**; visible image in 5E). Within cells, individual protein bodies (green) surrounded by lipid-rich areas (red) could be discerned. Calculation of component ratios is useful for cross-sample comparison as it accounts for sample section thickness variation; this comparison is not as reliable for pure peak integration visualization as presented in the RGB images.

**Table 2:** Canola composition analysis.

	% Oil	% Protein	% Phytate
NAM13	45.83	23.69	4.35 ± 0.30
NAM45	31.53	33.26	0.19 ± 0.02

To examine elemental distributions in canola seed with varied phytate content, XRF reconstructed images from adjacent sections of the same seeds were collected. XRF mapping at SXRMB highlighted elemental differences between the NAM13 and NAM45 genotypes (**Figures 5F, G**). RGB images of Cd (red), P (green) and Ca (blue) showed primary distributions of Cd and Ca in the seed coat in high (**Figure 5F**) and low (**Figure 5G**) phytate genotypes. P was distributed throughout the seed tissues in both genotypes however, in the low phytate genotype the distribution appeared consistently homogeneous. This was better illustrated by XRF images showing P distribution without other elements (**Figures 5H, I**). The high phytate genotype (**Figure 5H**) showed high levels of P in the embryonic axis, with mid-range levels in the inner lobes of the cotyledon, and low levels of P in the outer cotyledon lobes. In contrast, the low phytate genotype (**Figure 5I**) showed high levels of P throughout the seed. These MidIR and XRF images showed co-localization of P and protein within the seed.

Given the phytate status of the canola NAM genotypes, an important consideration was to identify P independently of protein, to ultimately identify P in the form of phytate. P K-edge XANES of select regions of the high phytate (**Figure 5J**) and low phytate (**Figure 5K**) genotypes confirmed the presence of P, although no speciation difference was detected (**Figure 5L**). A XANES measurement of the P signal was also important for subsequent spectromicroscopy (SM), to confirm the presence of P, to determine the energy to be used for on/off-resonance measurements, and to minimize beam time.

XRF at the SM beamline reveals element distributions with nano-scale resolution. Mapping of P within the cotyledons of canola (Topas; **Figures 6A, C**) showed the presence of punctate deposits of P within defined structures, consistent with globoids within protein storage vacuoles. These deposits were also identifiable in the NAM45 low phytate genotype, however, the distribution was more diffuse and indicative of fewer crystallized phytate deposits (**Figures 6B, D**). Semi-quantitative measures of P content may be obtained by counting the pixels above background that represent the P signal, within defined regions of interest, in this case whole cells (red circles). When normalized for cell size, Topas (**Figure 6C**) had 5648 counts/1000 pixels, while the low phytate line (**Figure 6D**) had 4521 counts/1000 pixels. These data support a stronger, localized P signal within subcellular domains of Topas canola cotyledon cells (**Figure 6A**) than the P in a low phytate canola genotype (**Figure 6B**). Thus, the distribution of P within the putative protein storage vacuoles made possible by the high-resolution mapping of elements at the SM beamline is critical for the interrogation of phytate deposition within these genotypes.

The power of cross-beam multi-modal imaging for correlating structural features with compositional traits is demonstrated with the use of the same canola seed (**Figure 7A**) for imaging at multiple beamlines. A false-coloured slice through an SR-µCT image (**Figure 7C**) can be identified from a 3D rendering of a whole canola seed (**Figure 7B**; **Supplementary Video 4**). After freezing and cryo-sectioning of the SR-µCT imaged seed, the same slice can be directly compared to those imaged by light microscopy (**Figure 7D**), MidIR (**Figure 7E**) and XRF (**Figure 7F**) spectroscopy. SR-µCT images show detailed morphology of the cellular composition of the seed (as shown in the pan through in **Supplementary Video 5**), which can be critical for the interpretation of reconstructed chemical images with lower resolution or limited compositional differences. When imaged with MidIR, the slice of interest contained an embryonic axis rich in protein (green), cotyledons rich in lipids (red) and a seed coat rich in carbohydrate (blue; **Figure 7E**). XRF spectroscopy in micro-mode (5 µm resolution) showed the distribution of Ca (red), Fe (green) and Zn (blue) within an adjacent section of the same seed (**Figure 7F**). This demonstrates that the embryonic axis contained high levels of Zn while the seed coat and the cotyledons were rich in Ca. Iron in this canola seed had a discrete denticulate localization within the cotyledons when observed by XRF spectroscopy, which could be precisely mapped to the vasculature region of the cotyledons, and a central domain in the radicle using SR-µCT datasets. With these complimentary SR approaches, observations made in one dataset could be informed by another.

These synchrotron chemical imaging techniques proved to be valuable for identifying the overt differences between dicot and monocot species. MidIR scans of a mature wheat seed (or grain) (**Figure 8**) in a region without (**Figure 8A**) and with (**Figure 8B**) the embryo, followed by a diagrammatic rendering with noteworthy anatomic features labelled (**Figure 8C**), highlight that the endosperm was predominantly carbohydrate rich, the cotyledon (also called scutellum) was protein- and lipid-rich, and the embryonic axis was primarily comprised of protein. The pericarp was carbohydrate-rich but also had a prevalent localized lipid component (**Figure 8A**). The seed coat was rich in lipids and the underlying aleurone and sub-aleurone endosperm layers were protein-rich. An XRF (**Figure 8D**) false-coloured image revealed Ca (red), K (green) and Mn (blue) distribution is the adjacent section of the same wheat seed. These data showed prominent K in the bran (consisting of pericarp, seed coat and aleurone) and embryonic axis, preferential presence of Mn in association with the endosperm, and Ca in the embryo (**Figure 8D**).

## 4 Discussion

Widespread availability of vast genomic resources has ushered in a “post-genomics” era for research in the life sciences, in which a focus on integrating genetic and phenotypic data to functionally analyze and decipher biological phenomena is forefront (Tuggle et al., 2024). Synchrotron imaging offers an array of tools for uniquely accessing phenotypic traits, including comprehensive measures of an organisms’ internal architecture and metabolic composition. Here, we featured a panel of seed datasets analyzed at the Canadian Light Source (CLS) to demonstrate the potential for synchrotron technology to reveal the inner workings of seeds. Methods detail seed preparation possibilities for imaging at five synchrotron beamlines (including BMIT-BM, MidIR spectroscopy, BioXAS-Imaging, SXRMB, and SM). Data presented demonstrate the value of imaging tools available at each beamline for studies of seed embryo, endosperm and seed coat anatomy, development, physiology and biochemistry. While each beamline can inform different questions, the use of multimodal imaging with synchrotron technology is not common in the literature, especially in the field of plant science. Indore et al. (2023) demonstrate the use of µCT, MidIR and XRF to determine changes in barley seed during storage, however, where multimodal SR imaging exists, it is generally limited to CT and XRF (e.g Varga et al., 2020; Calo et al., 2022; Nakhforoosh et al., 2024). We demonstrate the power of combining synchrotron hard and soft X-ray modalities to resolve phenotypic and metabolic complexities and correlate structural and chemical datasets within the same seed. Together with genomic insights, synchrotron technologies support comprehensive seed analyses and open doors to informed crop improvement.

SR-µCT imaging (using BMIT-BM beamline) provides superior penetration into specimens and is ideally suited to the study of whole seeds. SR-µCT imaging reveals the complex internal anatomy of seeds with micron and submicron resolutions, allowing cellular boundaries and subcellular features to be distinguished. Further, 3D volume rendering and modeling enable quantitative measures of complex forms to be made accurately, while providing novel views of the spatial relationships and interfaces that link seed subcompartments. SR-µCT imaging allows the extent of necrosis due to bacterial penetration into a seed to be distinguished from surrounding networks of seed vasculature (**Figure 2**). Localization of bacterial fruit blotch pathogen within watermelon seeds (case study 1) is dependent on the method of infection with embryonic localization resulting from pistil penetration (Dutta et al., 2012). Penetration into the embryo (as seen in the current study), as compared to perisperm-endosperm layer localization following ovary pericarp infection, is associated with increased survival of the bacteria following desiccation and exposure to seed treatments such as peroxyacetic acid and chlorine gas (Dutta et al., 2016). While seed infection status and seed treatment efficacy can be assessed using seedling grow-out assays, SR-µCT allows for visualization of seed health and could serve to increase understanding of pathogenesis and to aid the development of better treatments to control outbreaks. This study also provides a novel proof of concept for segmentation of necrotic regions of seed tissue following infection. Networks of air gaps can similarly be mapped with SR-µCT, to assess the impacts of prolonged dormancy and storage on seed viability (Cloetens et al., 2006). SR-µCT imaging can be applied to immature seeds to gain access to internal developmental processes that occur in each subcompartment. In pea (case study 2), this included precise volumetric size measures for all seed subcompartments and apoplastic spaces, the stage of embryonic development, the degree of endosperm degeneration, and the 3D architecture of inner seed coat layers not present in mature peas (**Figure 5**; Weber et al., 2005). 3D rendering at this resolution provides complete seed information thus allowing interrogation of relationships not possible with traditional 2D light microscopy. Perhaps most advantageous is the ability the BMIT-BM beamline offers to non-destructively examine mature seeds, bypassing cumbersome preparation efforts (**Figure 1**) and maintaining the possibility for post-imaging seed analyses, and even seed germination.

SR-µCT imaging presented here employed hard X-rays with 12.6 – 40 keV (BMIT-BM) energy range which is sufficient to visualize the internal structures of valued crop seeds in Canada. However, harder X-rays (available with high-energy BMIT-ID, with 28 – 140 keV photon energies) can accommodate SR-µCT imaging of large seeds and plant structures, as demonstrated for wheat root architecture (**Supplementary Figure 2**) as well as high density materials. In biological materials, the majority of elements have a low atomic weight, therefore only weakly absorb X-rays and make it difficult to differentiate tissues and structures using absorption-contrast imaging. Phase retrieval in seeds can improve visualization of embryonic cotyledons (**Figure 2**) and supply information regarding particle size and distribution based on histogram peak shapes and shifts (**Figure 3**). Phase retrieval is necessary in some instances such as mapping complex networks of vasculature (**Figure 2**) where conventional attenuation/absorption contrast limits feature demarcations. The use of SR-µCT imaging of seeds, in combination with targeted solvent extractions, can additionally provide indications of chemistry that is localized within tissues and cells (**Figure 3**).

The ability to obtain µCT images from plant tissues is not unique to synchrotron radiation as laboratory X-ray scanners can also perform this task (Karahara et al., 2023). A recent report indicates that the use of lab-based scanners is approaching that of synchrotron-based scanners (Zwanenburg et al., 2021). However, synchrotron-based scanners offer a number of key advantages, including faster scan times that can protect sample integrity and reduce motion artifacts for hydrated samples, adjustable propagation distances for increased flexibility and control in phase contrast imaging, and energy tunability which allows for optimization of contrast (Garcea et al., 2018; Kopittke et al., 2018). Phase retrieval is possible with some lab-based scanners but, the methodology required reduces the photon flux of these instruments, thus, extending scanning times (Quenot et al., 2022). The parallel, monochromatic, brighter beam with SR also results in a better signal to noise ratio than the divergent, polychromatic beam of lab scanners, yielding better image quality in images of equal resolution. The energy tunability of synchrotron light also provides an advantage in elemental analysis over lab-based instruments, as SR X-ray absorption spectroscopy can probe distinct species (Kopittke et al., 2018).

Access to synchrotron facilities is a potential hurdle, especially if time is required on multiple beamlines. However, the assistance of expert beamline staff in drafting proposals and designing experiments as well as the subsequent peer-review process (https://www.lightsource.ca/users/getting-started/peer-review-process.php) governing access to beam time ensure high quality research is awarded time. The community of practice further opens access to users who are less familiar with these technologies and those who can’t afford to purchase, develop and maintain these technologies. While lab instruments might be more accessible to some researchers, this is not the case for all and access to lab instruments for multiple imaging modalities is likely more rare.

Synchrotron-based imaging and spectroscopy are immensely powerful tools for the study of chemistry in heterogeneous biological materials. The techniques employing hard and soft X-rays in this study, together with Mid-IR spectroscopy, provide broad detection capacities for a range of plant metabolites. These synchrotron imaging tools provide spatial context to biochemical analyses, allowing metabolic landscapes inside seeds to be mapped with micro- and even nano-scale resolutions. Seed nutrition traits can therefore be assigned specifically to the embryo, endosperm or seed coat. Using MidIR spectroscopy, the distribution of protein, lipid and carbohydrate was revealed in protein-rich pea (case study 2; **Figure 4**), oil-rich canola (case study 3; **Figures 5**, **7**), and carbohydrate-rich wheat (**Figure 8**) seeds. The use of canola seeds with contrasting phytate levels illustrates the value of spatially-resolved analytics for informed seed variety comparisons. The synchrotron-based imaging tools applied in case study 3 resolved the spatial relationship between oil and protein within the embryo. The sequestration of macromolecules within canola seed embryos is expected based on the presence and organization of protein storage vacuoles and oil bodies. These protein storage vacuoles are the expected location of the phytate crystals in canola seeds. In pea, spectra from seed coat sclerenchyma and parenchyma cell layers of the immature seed coat, as well as features within the embryo (including the radicle and cotyledons) were readily distinguished by distinct macronutrient profiles (**Figure 4**). The contrasting chemistries of wheat grain subcompartments (**Figure 8**) were particularly marked, with stark distinctions in the macronutrient reserve. These results highlight the radial gradient of the starchy endosperm with the sub-aleurone layer being rich in protein and relatively limited in starch content (for review see Shewry et al., 2020). The addition of correlative µCT could aid in the identification of cell types with these different distribution patterns throughout the seed.

Conventional MidIR spectroscopy provides a valued overview of the macronutrients within seed subcompartments, resolving organ and tissue chemistries, and prominent cell layers within these subcompartments. IR spectroscopy’s detection potentially extends far beyond the macronutrient-focused seed analyses presented here to a range of organic macromolecules (see reviews by Schulz and Baranska, 2007; Dokken et al., 2005; Türker-Kaya and Huck, 2017; Cozzolino, 2012; Abidi, 2021). Of particular interest to plant research, cell wall polysaccharides such as cellulose, hemicelluloses, and pectin (Lahlali et al., 2015; Kumar et al., 2016; Pereira et al., 2018), biopolymers including suberin, lignin, and cutin (Zeier and Schreiber, 1999; Moore and Owen, 2001; Yan et al., 2009; Tanino et al., 2017), and diverse specialized metabolites and natural products (such as phenolic compounds, flavonoids, alkaloids, glucosinolates, essential oils, terpenes and glycosides; see review by Cozzolino, 2009) can be resolved by IR spectroscopy. The majority of MidIR spectroscopy chemical imaging presented here employed a conventional IR source, rather than bright synchrotron light. Synchrotron IR spectroscopy offers improved signal-to-noise over conventional IR, which improves lateral spatial resolutions for spectra that can be distinguished at the cellular level (**Figure 5**). Sub-diffraction limited IR imaging techniques such as optical photothermal IR (O-PTIR) and nano-scale IR techniques offer additional IR tools for chemically analyzing biological materials with sub-micron and nanometer resolutions, respectively.

Micronutrient maps across seeds can be captured in parallel with macronutrient maps for comprehensive analysis of seed nutrient compositions and localization patterns. XRF employing hard X-rays and soft X-rays was used to illustrate the range of detectable elements that can be mapped across seeds. The CLS BioXAS-Imaging beamline is well suited for XRF elemental mapping of biologically relevant elements (e.g. K, Ca, Mg, Mn, Fe, Zn, Cu) as shown in **Figures 4**, **7** and **8**. Soft X-ray XRF performed at the CLS SXRMB increases the range of detectable elements, being ideally suited to lighter atomic weight elements (e.g. Si, S, P, Cl, K, Ca) as featured in **Figure 5**. SXRMB detection of some heavier elements, such as Cd, can be accessed through their L-edge emission.

Soft X-ray spectromicroscopy (SM) allows imaging elements with lower atomic weights (e.g. C, N, O, Na, Mg, Si, P) than SXRMB and BioXAS, and provides spatial resolution to probe composition of fine subcellular structures within a seed. Here, we illustrated the value of SM for comparing canola seeds with contrasting phytate levels (**Figure 6**). SM was able to distinguish punctate P deposits independent of the protein content of the cell. These are indicative of phytate crystals in a high phytate genotype as compared to a generalized P localization in the low phytate line. Using XRF imaging with SM, it is also possible to collect 3D data as demonstrated by Kim et al. (2006). While 3D XRF can provide important spatial context for understanding elemental distribution in samples, there are a number of challenges with 3D XRF in biological tissues. The sample must be stable under extended scan times and 3D rendering for low atomic weight, biologically relevant elements is limited to thin samples due to self-absorption of the XRF signal. Self absorption is also an important consideration for 2D XRF as highlighted by Pushie et al. (2022). In addition to informing elemental distributions in seed cells, SM can be used to determine differences in elemental speciation with high spatial resolution. XANES of select elements (P at the SXRMB beamline, **Figure 5**; zinc at the BioXAS-Imaging beamline, **Figure 4**) demonstrates this capacity. XANES spectra for Zn in developing pea seeds show different species of Zn between seed tissues. With the availability of appropriate standards, the specific species could be identified. XANES at SXRMB was not able to differentiate P species at this resolution, however, in general, speciation detection of P in biological samples is problematic as there are multiple species in a complex matrix and the K-edge spectra have limited distinguishable features (Schmieder et al., 2020). Greater differentiation of spectra is possible using P L_2,3_-edge XANES however, the concentration of P required is significantly greater than that for detecting P with its K-edge (Kruse et al., 2009).

The ability to map metabolic compositions to the seed coat, embryo and endosperm allows spatially resolved insights into macronutrient and micronutrient compositions in seeds. We highlight the flexible and complementary nature of synchrotron imaging tools for seed research. Cross-platform synchrotron imaging, as presented here, strengthens understanding of structural and compositional data. Although technically challenging, obtaining correlated datasets for the same seed across beamline techniques is increasingly feasible. Semi-correlative data presented used neighbouring sections for macro- and micronutrient profiling in pea (**Figure 4**), and correlative microscopy across synchrotron platforms deciphered macro- and micronutrient compositions and structural landscapes at the same locale within a single canola seed (**Figure 7**). XRF highlights the abundance of iron in the putative endodermal cells surrounding the vasculature (**Figure 7F**) as previously demonstrated in arabidopsis (Kim et al., 2006; Grillet et al., 2014) and *Brassica napus* (Ibeas et al., 2017). This demonstrates the value of imaging across beamlines, as the cell types associated with iron signal from XRF could be directly correlated with cell types in the same seed in SR-µCT images. This correlative imaging approach provides novel information not gleaned from either beamline used individually. Continued efforts to support correlative microscopy across synchrotron beamlines promise to facilitate direct comparisons between structural and compositional imaging datasets, for more complete understanding of the physical and chemical properties that function within heterogeneous living systems. By revealing spatiotemporal complexities of the composition and architecture of embryos, endosperm and seed coat, with opportunities for non-destructive and correlated imaging, these methodologies have immense value potential for seed biologists, seed bank curators, and breeders alike.

The computational resources and expertise required to analyze large SR-generated data presents a considerable limitation to the use of SR technology. Enhancements in analysis pipelines, AI and machine learning algorithms promise to reduce bottlenecks in most existing SR data analysis pipelines. For example, Albers et al. (2024) describe a high-throughout tomography platform with an automated data processing pipeline that provides a 3D reconstructed data set less than two minutes after the X-ray scan is complete. Segmentation of 3D µCT data is an additional bottleneck, requiring time, experience and extensive computational resources. Advances in deep learning have provided tools such as Biomedisa (Lösel et al., 2020) and Google’s Colaboratory web application (described by Rippner et al., 2022) to increase the speed, computational efficiency and accessibility of segmentation. Modules available in Quasar (Toplak et al., 2021) include machine learning algorithms for the analysis of spectroscopy data and are accessible to non-computer experts. Excellent reviews of these algorithms and advances in this space are provided by Meza Ramirez et al. (2020) and An et al. (2022). As novel tools in this space continue to emerge, accessibility of SR-based technology will become increasingly feasible for researchers. Growth of SR-guided research could enable the integration of anatomic and biochemical phenotypes with the genotypic and transcriptomic datasets more commonly available, to enhance critical understanding of the fundamental biological processes and the mechanisms that underlie seed trait variation. Developments in the field of SR-driven research suggest a bright future for synchrotron-informed research in plant sciences.

## Data Availability

The original contributions presented in the study are included in the article/**Supplementary Material.** Further inquiries can be directed to the corresponding author.

## Supplementary material

Supplementary Figure 1 SR-µCT imaging of a developing pea seed showing cellular structure of the seed coat and embryo **(A)** and the localization and structure of the 3D rendered embryo within the seed coat **(B)** and at different orientations **(C, D)**. Scale bar 800 µm.

Supplementary Figure 2 SR-µCT imaging of wheat roots in soil, produced at the Canadian Light Source BMIT-ID beamline. Plants were grown in Sunshine Mix #3 in 7.5 cm diameter customized rhizotrons. The top ~15 cm of the 120 cm rhizotron was imaged. Roots were manually segmented from SR-µCT slices using Avizo software **(A)**; red and green are examples of segmented primary roots). Three-dimensional volume rendering of primary roots is shown in **(B)** and secondary roots are shown with the primary roots in **(C)**.

Supplementary Video 1 Sequential slices through SR-µCT imaged watermelon (*Citrullus lanatus*) seeds prior to segmentation. A healthy seed is seen in the left panel while the right panel shows a seed infected with bacterial fruit blotch *Acidovorax citrulli*.

Supplementary Video 2 SR-µCT of bacterial fruit blotch infection (*Acidovorax citrulli*) necrosis (blue) as occurring in sequential slices of a watermelon (*Citrullus lanatus*) seed. See **Figure 2D** for a single slice (not coloured) selected from this image series.

Supplementary Video 3 Three-dimensional rendering of watermelon (*Citrullus lanatus*) seed showing bacterial fruit blotch (blue) produced by *Acidovorax citrulli* and seed vasculature (red) following SR-µCT. See **Figure 6C** for a representative plane from this 3D view.

Supplementary Video 4 Three-dimensional rendering of a canola (*Brassica napus*) seed SR-µCT image highlighting the location of the false-coloured slice shown in **Figure 7C**.

Supplementary Video 5 High resolution, sequential slices through an SR-µCT-imaged canola (*Brassica napus*) seed, prior to segmentation.
