# Supplementary figures and images for: Applications of synchrotron light in seed research: an array of x-ray and infrared imaging methodologies
Source: Front Plant Sci. 2025 Feb 17;15:1395952. doi: 10.3389/fpls.2024.1395952 (PMC11873090; doi:10.3389/fpls.2024.1395952)

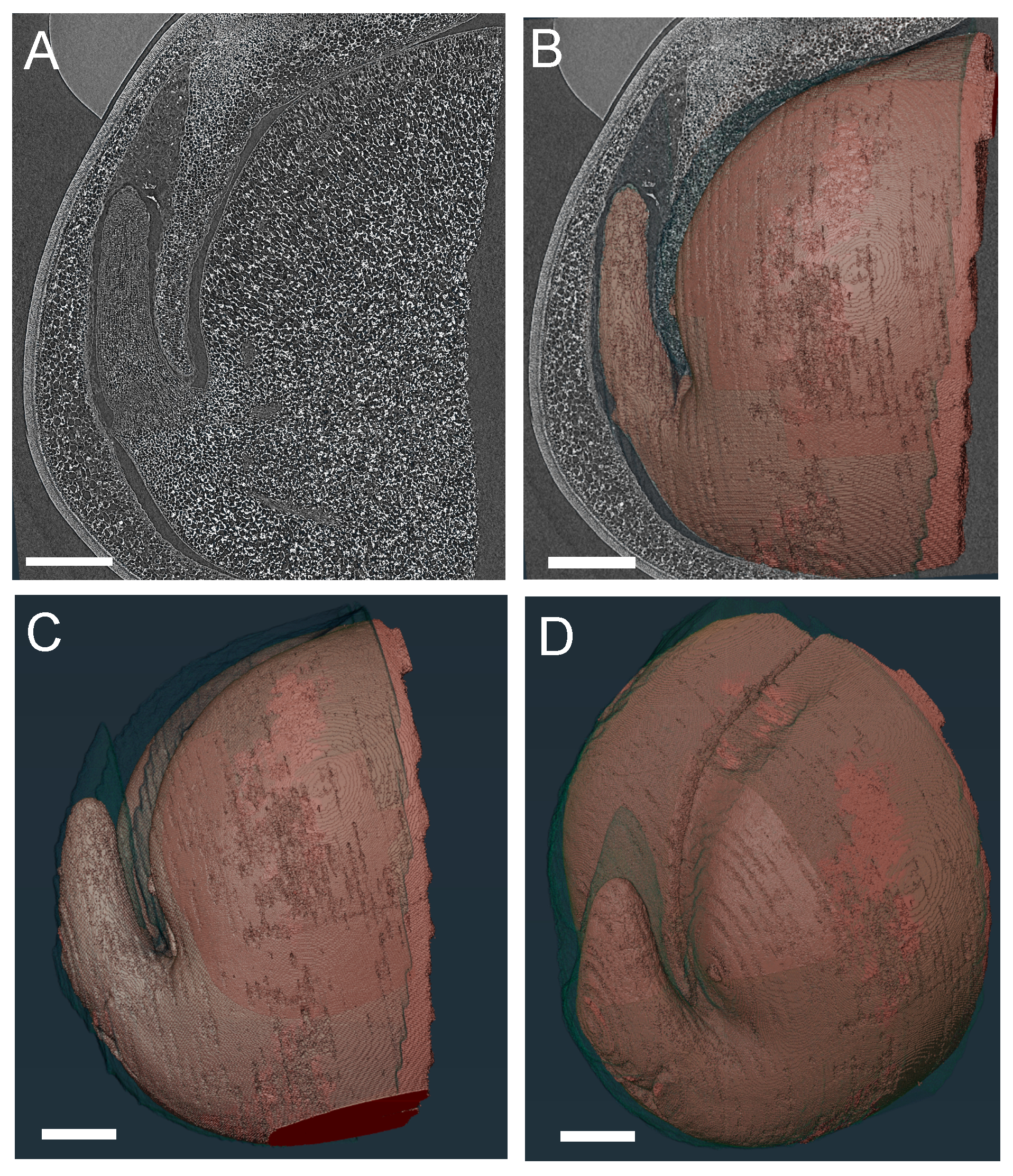

Supplement: Supplementary Figure 1 — SR-µCT imaging of a developing pea seed showing cellular structure of the seed coat and embryo (A) and the localization and structure of the 3D rendered embryo within the seed coat (B) and at different orientations (C, D). Scale bar 800 µm. [file Image1.jpeg]

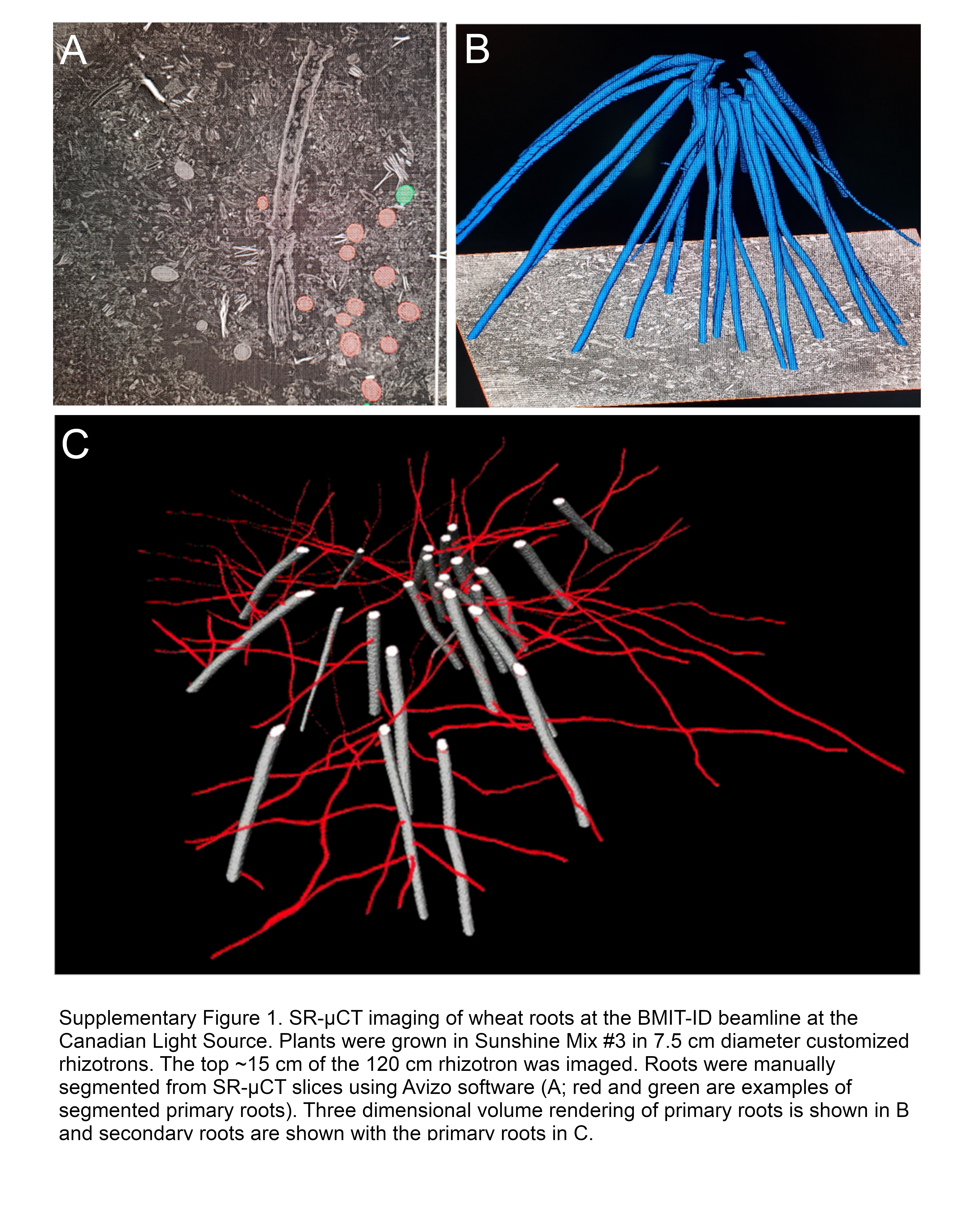

Supplement: Supplementary Figure 2 — SR-µCT imaging of wheat roots in soil, produced at the Canadian Light Source BMIT-ID beamline. Plants were grown in Sunshine Mix #3 in 7.5 cm diameter customized rhizotrons. The top ~15 cm of the 120 cm rhizotron was imaged. Roots were manually segmented from SR-µCT slices using Avizo software (A); red and green are examples of segmented primary roots). Three-dimensional volume rendering of primary roots is shown in (B) and secondary roots are shown with the primary roots in (C). [file Image2.jpeg]
