# Supplementary material for: Applications of synchrotron light in seed research: an array of x-ray and infrared imaging methodologies
Source: Front Plant Sci. 2025 Feb 17;15:1395952. doi: 10.3389/fpls.2024.1395952 (PMC11873090; doi:10.3389/fpls.2024.1395952)
Supplement: Supplementary file 9 [file DataSheet1.docx]

Supplementary Methods

### Chemical fixation of immature pea seeds for SR-µCT imaging (BMIT-BM)

The protocol outlined in section (2.3.1) is provided below, with additional protocol details and suggestions.

- Use a dissecting stereo microscope and forceps to remove developing seeds from the parent plant without damaging the seed coat.
- Minimize time from dissection of seeds to chemical fixation (for SR-µCT) or cryo-preservation (chemical imaging).
- Recommended fixative solution: Glutaraldehyde diluted to a final concentration of 2% (v/v) in 25 mM PIPES buffer. Prepare fresh fixative solution on the day it will be used.
- PIPES buffer made with PIPES monosodium salt (MW 324.3), pH adjusted to 6.8 with 1 M filter sterilized NaOH (MilliporeSigma, Oakville, ON), is recommended for plant cells, although cacodylate buffer is an acceptable alternative. 25 mM buffer was used throughout because more concentrated buffers (>100mM) can cause plasmolysis. Stock solutions of concentrated PIPES buffer (200 mM) may be stored at 4˚C.
- For medium-large seeds such as pea, ensure complete penetration of fixative through the seed coat is possible. This can be facilitated by making a small cut through the seed coat with a fine, sharp razor blade (Double edge Personna razor blades, Electron Microscopy Sciences; Cedarlane Burlington, ON) to ensure complete fixation of the internal embryo. Alternatively, if volumetric quantification is not required, large seeds can be cut in half to facilitate fixation.
- Gently submerge the nicked seeds in freshly prepared fixative solution (2% glutaraldehyde solution in 25 mM PIPES buffer, pH 7.0) in glass scintillation vials. Plant material to fixative ratio should be 1:1000. Glass scintillation vials (OPTICLEAR™ borosilicate glass vials; Avantor, Mississauga, ON) recommended for fixative incubations can be 5 mL to 20 mL, depending on the size and number of seeds to be fixed.
- Fix at room temperature for 2 hours or overnight at 4˚C.
- If seeds do not sink, gentle vacuum infiltration can be used.
- Following fixation, remove the fixation solution, ensuring samples remain submerged. Replace with 25 mM PIPES buffer, pH 7.0 without glutaraldehyde. Repeat 5 times, to ensure fixative has been removed.

### Dehydration of immature pea seeds for SR-µCT imaging (BMIT-BM)

- Dehydrate samples in ascending ethanol; 30%, 50%, 70%, 95%, 100% with a minimum of 20 minutes incubation at room temperature between each exchange. Stop the dehydration at 70% ethanol and store at 4˚C if not proceeding directly to critical point drying. Ensure that seeds are always covered with liquid and not exposed to air.
- Repeat a minimum of three 100% ethanol exchanges for 20 minutes each.
- Continue the exchanges until colour is no longer leeching from the sample and ethanol solution is clear. For thick materials exceeding 1 cm in diameter, it is recommended to lengthen time between exchanges, and to allow seeds to remain in 100% ethanol for a minimum of 48 hours prior to critical point drying.
- Critical point dry the samples (Autosamdri®-931 Critical Point Dryer, Tousimis Research Corp, Rockville, MD in stasis mode; using 3 cycles) with solvent-substituted liquid CO_2_ as a drying agent (Carbon dioxide 3.0, Syphon, K-size “bone dry” (Linde, Saskatoon, SK). Place the drying chambers in 100% ethanol, and transfer samples from the glass vials to the drying chambers ensuring they are not exposed to air. Thoroughly dried samples should have no detectable ethanol odour.
- Store samples at room temperature in a sealed container away from light, with dust-free desiccant.
